# Plant-Based Alternatives Need Not Be Inferior: Findings from a Sensory and Consumer Research Case Study with Cream Cheese

**DOI:** 10.3390/foods13040567

**Published:** 2024-02-13

**Authors:** Sara R. Jaeger, David Jin, Christina M. Roigard

**Affiliations:** 1 Department of Food Science, Aarhus University, 8200 Aarhus, Denmark; 2 The New Zealand Institute for Plant and Food Research Limited, 120 Mount Albert Road, Auckland 1025, New Zealand; david.jin@plantandfood.co.nz (D.J.); christina.roigard@plantandfood.co.nz (C.M.R.)

**Keywords:** plant-based, cream cheese, liking, sensory, non-sensory, consumer, New Zealand

## Abstract

Reliance on animal foods must be reduced to improve planetary and human well-being. This research studied plant-based cheese alternatives (PBCA) relative to dairy cheese in a consumer taste test with 157 consumers in New Zealand. A case study approach used cream cheese (commercially available) as the focal product category (2 PBCA, 2 dairy) and implemented a multi-response paradigm (hedonic, sensory, emotional, conceptual, situational). “Beyond liking” insights were established, including drivers of liking (sensory, non-sensory) and sensory drivers of non-sensory product associations. Two consumer segments were identified, of which the largest (*n* = 111) liked PBCA and dairy samples equally (6.5–6.7 of 9). In this *PBCA Likers* cluster, the key sensory drivers of liking were ‘creamy/smooth mouthfeel’, ‘dissolves quickly in mouth’, and ‘sweet’, while a significant penalty was associated with ‘mild/bland flavour’. The non-sensory data contributed additional consumer insights, including the four samples being perceived as differently appropriate for 9 of 12 use situations, with PBCA being regarded as less appropriate. In the limited confines of this case on cream cheese, the findings show that PBCA need not be inferior to their dairy counterparts despite a general narrative to the contrary. Of note, the results were obtained among participants who were open to eating a more PB diet but were not vegetarian or vegan.

## 1. Introduction

### 1.1. Motivation for the Research

Transitioning to a diet that is dominantly plant-based (PB) will significantly benefit environmental well-being, biodiversity, welfare of farm animals, and human health [1,2,3]. Increasingly, consumers are embracing this shift and expecting dietary alternatives that support the PB transition. The development of PB alternatives to animal-based and animal-derived foods directly reflects this transition, and the present research is situated in this context. It focuses on PB alternatives to cheese, hereafter PBCA (i.e., PB cheese alternatives), because this product type could be a gateway category to support consumers’ PB transition. In a cohort of ~200 Dutch consumers who ate meat less than five times per week, cheese (34%) was listed as one of the top three common substitute foods after fish (79%) and eggs (49%) [4]. In a recent four-country consumer survey (USA, Australia, Singapore and India, *n* = 2494) by Cardello et al. [5], stated willingness to consume PB alternatives was highest for milk alternatives and lowest for fish alternatives. Willingness to consume was intermediate for two PB meat alternatives but lower than for PBCA. In other words, PBCA was the category of PB alternative foods that participants said they were most willing to consume, after PB milk alternatives. Although consumer segmentation was observed, the majority of participants tended toward higher rather than lower stated willingness to consume PBCA. 

Thus, it is not highly unrealistic to assume that a significant number of consumers could be willing to eat PBCA if products satisfy sensory quality, nutrition, price, and several other criteria that drive daily decisions about what to eat and drink [6]. In fact, it is possible that the lack of desirable product options in this category will hinder the PBdiet transition. In a survey of more than 582,000 people from 209 countries and territories who in 2021 took the “veganuary” pledge to be vegan for the month of January, 41% reported that cheese was the product they missed most [7]. This likely reflects that cheese plays some importance in many peoples’ diets because of its many varieties and their many uses [8,9].

With over 1000 different types of cheese available on the market, made from a wide range of animal milk [10], there is no shortage of sensory experiences for PBCA to attempt to replicate. However, the inherent challenge is that PB ingredients cannot directly replicate the sensory characteristics of dairy cheese [11,12,13,14]. This is key to challenges with consumer acceptance of PBCA (see below).

### 1.2. Research Objectives, Expected Findings and Empirical Overview

Building on the above and the still limited sensory and consumer science knowledge about PBCA, the present research was structured around four objectives, which in different ways explored issues linked to PBCA sensory and consumer acceptance (or lack thereof).

- Objective 1. To measure liking and provide a consumer-centric and extensive characterisation of PBCA through an empirical strategy that compared these to dairy cheese.
- Objective 2. To determine sensory and non-sensory drivers of liking.
- Objective 3. To determine sensory drivers of non-sensory product perceptions.
- Objective 4. To explore determine if consumer groups with different liking/disliking patterns for PBCA existed (i.e., consumer segmentation).

#### 1.2.1. Objective 1

The expectation based on previous research was that PBCA would be moderately or less liked, on average. This drew on past research suggesting that PBCA often have problems with some aspects of sensory quality [15,16,17]. For example, they can have sensory characteristics that are not typically associated with cheese [14], leading to dissatisfaction with the experienced sensory characteristics and unmet product expectations [18]. Studies that report liking data for PBCA confirm this [11,19], but also draw attention to an important fact—that some PBCA have liking scores [11] that are similar to those reported for cow’s milk cheeses [20,21,22,23]. Some studies exist where PBCA prepared with novel processing methods and/or ingredient combinations have been positively evaluated using small, convenience-based consumer samples [24,25]. These point to a future of increased consumer acceptability but may not represent the status quo. Pointke et al. [26] provide an example of PBCA acceptance among vegan consumers but also find that such liking scores, on average, are higher than those from consumers following omnivore and flexitarian diets.

Based on the literature cited above, the sensory product characteristics of PBCA and dairy cheese were expected to be different (Objective 1). These different product experiences (hedonic and sensory) would, in turn, lead to different emotional, conceptual, and situational use perceptions for PBCA and dairy variants, as suggested in studies with foods other than cheese [11,27,28] and supported by studies reporting on the sources of food emotions and conceptualisations, and linkages between these non-sensory product perceptions [29,30].

#### 1.2.2. Objective 2

PB alternative products that mimic the sensory properties of non-PB alternatives tend to be preferred by consumers [14,19,31], and for this reason, it was expected that the sensory drivers of liking for PBCA would be those that increase liking of dairy cheese. Since it has been suggested that more than 1000 types of cheese exist [32] with very different characteristics [9], sensory drivers of liking will be specific to different types of cheese. Nonetheless, they are likely to encompass multiple sensory modalities (appearance, aroma, taste, flavour, texture, mouthfeel, and aftertaste) [20,21,33,34,35]. This extends to cream cheese (PBCA and dairy) [18,22,36,37]. Although non-sensory drivers of liking have not been widely studied, it was expected that emotions with positive and negative valence would drive liking positively and negatively, respectively [29,38], and emotional activation/deactivation could contribute to product differentiation [39].

#### 1.2.3. Objective 3

The sensory drivers of non-sensory product experiences have also not been widely studied. The reason to study these directly flows from the now widely accepted mindset that sensory and consumer science needs to “go beyond liking” [40,41]. Product experience is about much more than the sensory product characteristics, and it is important to address these, for example, by adopting a multi-response empirical strategy to measure emotional product associations [42], product conceptualisations [30], situational use characteristics [43], attitudes and behavioural intentions [44]. Given the scarcity of related past research, expected findings could not be clearly stated. However, it seemed probable that emotional associations with positive valence would be linked to sensory characteristics associated with positive hedonic experiences, and *vice versa* [45]. A decrease in emotional activation could be expected for weak, bland, and low flavour strength according to Jaeger et al. [46]. Regarding product conceptualisations, sensory characteristics typically associated with dairy cheese were expected to be perceived as familiar/traditional. Increased situational uses would be linked to sensory characteristics associated with positive hedonic experiences. At a more general level, linkages between desired sensory characteristics and positive, familiar, and versatile non-sensory product associations were expected to help overcome barriers consumers may experience in adopting PB alternative foods [47]. A recent example with yoghurt (PB alternatives, dairy, and PB–dairy blends) [48] illustrated that linkages exist in consumers’ minds between sensory characteristics and sustainability concepts with positive drivers of the latter including ‘white colour’, ‘sweet’, ‘even/smooth’ appearance and texture, ‘oat-like flavour’ and ‘coconut flavour’. ‘Dairy flavour’, on the other hand, evoked conceptualisations that were related to ‘traditional’ and ‘authentic’ and elicited negative sustainability concepts that were related to ‘resource intensive’, ‘commercially farmed raw materials’, and reduced conceptualisation of ‘animal friendly’.

#### 1.2.4. Objective 4

Consumer segmentation based on product liking was expected to exist. Considering that individual differences in preference are described for many foods and beverages [49] including dairy-based cheese [21,33,50] and PB alternative foods [51,52,53], interest in the present research was centred on the relative size of segments that liked, rejected, or partially accepted PBCA. Such knowledge is relevant because it helps to determine the magnitude of the change that has to occur for the consumption of PB alternative foods to become the norm rather than the exception.

#### 1.2.5. Case Study and Empirical Approach

Because it is not possible to cover the entire cheese category in a single study, a case study approach was employed as the research strategy. Cream cheese was selected as the focal category, but others could have been selected too. As a category, cream cheese originated in the USA in the 1870s [54]. It is a fresh (or unripened) cheese produced from a blend of milk and cream. The fat content (typically a minimum of 25% in dry matter) combined with a high moisture content [55] is critical for the characteristic soft and spreadable texture of cream cheese [56]. Lactic acid fermentation provides slight acidity and a “diacetylated taste and smell” [37,57]. Traditionally, cream cheese was a plain product with no additional flavourings, but nowadays, multiple flavour variants exist, particularly in the USA, as well as texture variants (firm, soft, airy) [58]. Largely a response to the growing consumer demand for healthier products, cream cheese with a lower fat content have also been formulated [22]. However, techniques for achieving fat reduction tend to result in texture and flavour changes that hinder consumer acceptance [22,57]. Non-dairy (or PB) alternatives to cream cheese also have an extended history, with reports of soy variants being produced in the USA between 1911 and 1970 [59].

Market data for PB alternatives to cream cheese are sparse but suggest that the category is currently very small, and this is within the PBCA category, which, compared to PB alternatives to meat and milk, is already modest [60]. Nonetheless, market projections point to growth. An estimated market value in the USA of USD 45 million in 2019 grew to USD 129 million in 2022, and there are projected estimates of USD 300 million by 2032 [61]. Beyond Western markets, growth is expected for China [62], where cream cheese is gaining some popularity in, for example, tea macchiatos and mooncakes.

To achieve the research aim and address the four objectives, a consumer taste test was conducted. Specifically, 157 consumers assessed four samples of cream cheese, of which two were dairy (cow’s milk) and two were PBCA. A trade-off was required between the number of samples, the number of consumers, and the extensiveness of evaluations for each sample, leading to emphasis being placed on the latter of the three criteria. That is, each sample was extensively described to facilitate insights relating to Objective 2 (sensory drivers of liking) and Objective 3 (sensory drivers of non-sensory product perceptions). Compared to Objectives 1 and 4, these two objectives were regarded as providing the most novel scientific insights.

## 2. Materials and Methods

### 2.1. Participants

The 157 participants were New Zealand (NZ) adults with varied socio and economic characteristics who lived in the Auckland region (Table 1). They were listed, by voluntary agreement, on a proprietary database of a professional recruitment firm and were willing to be contacted about participation in product taste tests. All were regular consumers of cheese (monthly or more frequently) and expressed an interest in reducing their meat and dairy consumption (i.e., it was an exclusion criterion to answer ‘no’ to the statement ‘I am not concerned about reducing my meat or dairy intake’). All participants confirmed willingness to taste cheese (dairy and PBCA).

### 2.2. Samples and Sample Presentation

#### 2.2.1. Samples

**Figure 1:**
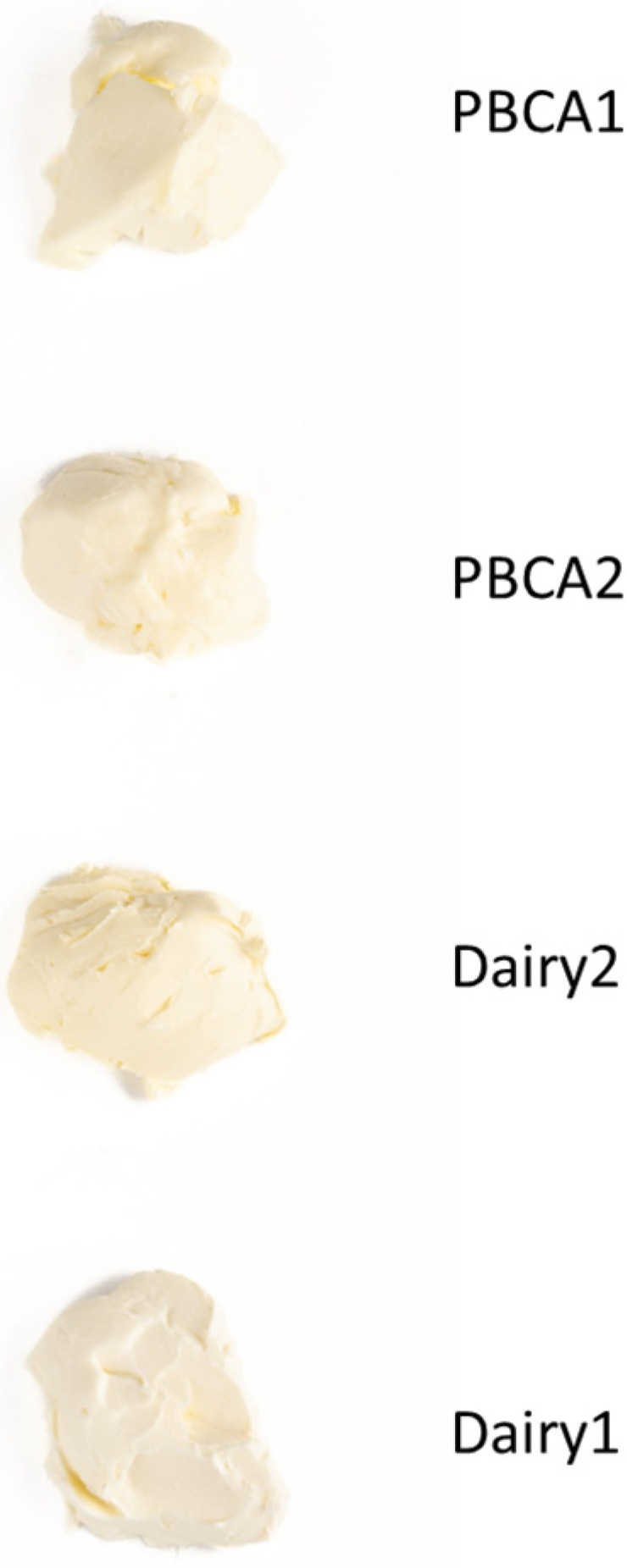
Images of four samples used in the study. Two were plant-based alternatives to cream cheese (PBCA) and two were dairy cream cheese (cow’s milk).

Four samples were included in the study. Two were dairy cream cheeses (cow’s milk) (Dairy1 and Dairy2), and two were PBCA (PBCA1 and PBCA2) (Figure 1). The samples were commercially available in NZ and from different brands. The PBCA samples were coconut oil-based, in line with the dominance of this ingredient in this product category [63]. At the time of the study, these were the only PBCA available in major retail outlets in the cream cheese product category. Additional information about the samples—nutritional composition, ingredients, and average price—can be found in the Supplementary Material. The samples were used as purchased, and no modifications were made before serving to consumers.

Cream cheeses are widely available in NZ retail outlets, and it was possible to obtain both PBCA and dairy variants that were “unflavoured” (or “natural” flavoured) in the sense that additional ingredients had not been added (e.g., herbs, spices). The decision to restrict the samples to a single flavour variant (“natural”) was made to increase the likelihood of differences between PBCA and dairy cheese being uncovered without undue influence from variations in flavour.

#### 2.2.2. Sample Preparation and Presentation

The samples were prepared in the mornings on the days of testing and were then covered and stored (~4 °C) until needed. They were removed from the refrigerator and served to participants within 20 min. and at approximately room temperature which aligned with Wendin, Langton, Caous, and Hall [57] and Foguel, Neves Rodrigues Ract, and Claro da Silva [22]. The serving size (approx. 10 g) allowed for re-tasting. Each sample was accompanied with a piece of ~5 cm breadstick (Ghiotti, Modea Srl, Roma, Italy). The samples were presented in 60 mL transparent glass bowls (odour-free) labelled with randomised numerical codes (Supplementary Material has image).

### 2.3. Responses by Consumers to Tasted Samples

Directed by Objectives 1 to 3, the test ballot was in five sections. Overall acceptability was the first responses and provided on a 9-point fully labelled category scale with 1 = ‘dislike extremely’ and 9 = ‘like extremely’ as end-point anchors.

Second to be elicited were sensory product perceptions. These were obtained using a CATA (check-all-that-apply) question with 12 descriptors (listed in full in Section 3). The terms covered appearance, taste, flavour, texture, and mouthfeel and were based on pilot work and the extant literature [22,55,57,64,65].

The third part of the ballot pertained to emotional associations elicited by the sample. These were obtained using 12 pairs of emotion words (listed in full in Section 3) from the valence × arousal circumplex-inspired emotion questionnaire [66]. The valence dimension was spanned between the word pairs ‘happy/satisfied’ (positive) and ‘unhappy/dissatisfied’ (negative), while the arousal dimension was spanned between the word pairs ‘passive/quiet’ (deactivated) and ‘active/alert’ (activated). A CATA question list format was used.

A conceptual CATA question with 14 terms (listed in full in Section 3) was the fourth ballot section. The terms were identified with input from the literature relating to conceptual profiling and PB alternative products [41].

In the fifth part of the ballot, a CATA question with 12 statements (listed in full in Section 3) captured perceived situational appropriateness. The statements were developed by the authors and based on pilot work and cookbooks [67].

### 2.4. Data Collection

The study was conducted at The New Zealand Institute for Plant and Food Research Limited (Auckland), where participants (in groups of 18 people) during October 2023 attended one 75 min. research session.

Monadic sample presentation was used, with all parts of the ballot being completed before presenting the next sample. Participants had to taste the sample before answering the first question and retaste as needed as they progressed through the ballot. Within CATA questions, the presentation order of terms was balanced both across samples and participants.

Compusense Cloud software (v23, Compusense Inc., Guelph, ON, Canada) was used for data acquisition. Participants were seated in standard sensory testing booths with white lighting for sample assessment. A 30–45 s break between samples helped to manage lab logistics and mitigate sensory fatigue. Water and plain crackers (Arnott’s original water crackers, NZ) were available throughout the test for palate cleansing. The temperature in the booths was between 20 and 22 °C.

After completing the cream cheese tasting task, participants completed another tasting task with cheese (not considered further) and a questionnaire with questions relating to cheese liking and frequency of consumption. A few additional tasks without relevance to the stated research objectives were then completed (not considered further). Participant profiling questions were asked as the last part of the research session.

### 2.5. Data Analysis

All analyses were performed in XLSTAT [68]. Unless stated otherwise, a 5% level of significance was used for inference testing.

Sample differences in mean hedonic values (Objective 1) were assessed using a generalised linear model (sample as fixed factor, consumer as random factor) and Tukey’s HSD for *posthoc* tests. Means and standard deviations were calculated for each sample.

For product characterisation (Objective 1), Cochran’s Q test was performed for each CATA term, using the Sheskin procedure for *posthoc* testing. Based on Vidal et al. [69], CATA term citation frequency was interpreted as a proxy for attribute intensity/strength. Separately for sensory, emotional, conceptual, and situational terms, Correspondence Analysis (CA) [70] was performed on the contingency table formed by crossing samples (rows) and CATA terms (columns) [71]. In all instances, the null hypothesis of independence between rows and columns was rejected subsequent to a chi-square test (*p* < 0.0001). Two-dimensional solutions, which accounted for >70% of variance in the data, were retained, and interpreted in the context of 95% confidence ellipses [72] around sample positions.

For each CATA term, penalty/lift analysis was performed to establish its mean impact on liking [71] (Objective 2). The significance of the mean impact was only tested when a threshold value of 10% citation frequency was exceeded.

Logistic regression was used to establish sensory drivers (independent variables) of non-sensory product associations (dependent variables) (Objective 3). Models were estimated for each of the dependent variables and interpreted only when significant at the 1% level according to the criterion of −2 log likelihood (-2LL). Odds ratios (OR) were used to express the effects of predictor variables [73]. When ORs > 1, there is an increased probability of selection of Y (dependent variable) when X (independent variable) is selected. When ORs > 1, there is a increased probability of selection of Y when X is selected. For example, OR = 2 means a doubling of selection probability. OR = 0.5 means a reduction of 50% in selection probability. Because the independent and dependent variables were binary, the estimated ORs could be interpreted as standardised effect size statistics, which enabled different OR values to be directly compared with each other.

Segmentation analysis based on sample liking scores (Objective 4) was achieved through hierarchical cluster analysis based on Euclidian distance and the method of Ward. Prior to cluster analysis, five participants were excluded because they gave the same liking score to all four samples. The number of clusters was determined using two criteria: scree plots and sample size of all retained clusters must exceed 40 people. The latter criterion followed from Moskowitz [74] who stated that stable estimates in sensory consumer research are often obtained from group sizes of 40–50 people. In each of the two retained clusters (n = 111, n = 41), all analyses described above were performed.

## 3. Results

### 3.1. Liking (Objective 1)

Considering all participants in the study (n = 157), the mean liking for the four cream cheese samples ranged between 5.9 and 6.8 on the 9-point hedonic scale (Table 2). These values meant that both the PBCA and the dairy samples were positively received (6 = ‘like slightly’ and 7 = ‘like moderately’). While cream cheese from cow’s milk was more liked than PBCA, the difference between the two product types was less than one hedonic scale point (6.8 vs. 6.0). The proportions of participants giving dislike scores (1 to 4 on the response scale) were lower for the dairy samples (8–11%) than for the PBCA samples (22–30%).

### 3.2. Product Characterisation (Objective 1)

**Figure 2:**
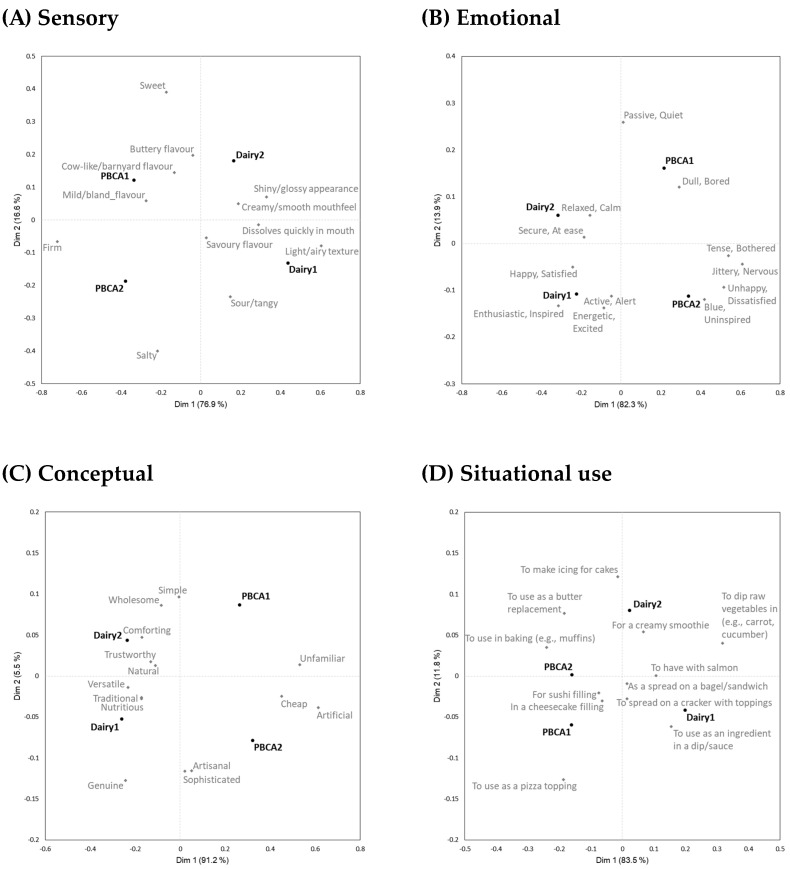
Results for the total sample of participants (n = 157), showing biplots of the first two dimensions after Correspondence Analysis of product characterisation data. (Descriptors are shown in grey and samples in black. To reduce visual clutter, 95% confidence ellipses around sample positions are not shown.

Product characterisation was extensive and comprised four parts. For each response type—sensory, emotional, conceptual, situational use—Table 3 shows the results from Cochran’s Q test (sample discrimination) and average term citation frequency across all samples. Figure 2 shows biplots of the first two dimensions after Correspondence Analysis for sensory, emotional, conceptual, and situational use data based on the total sample of 157 participants. To maintain visual clarity, 95% confidence intervals on sample positions are not shown, but can be found in Supplementary Material. Accompanying radar plots (by sample) for sensory, emotional, conceptual, and situational use data based on the total sample of 157 participants can also be found in Supplementary Material.

#### 3.2.1. Sensory

The samples were described using 12 sensory descriptors, of which 10 were discriminating (Table 3). While all four samples had distinct profiles (Figure 2A) (see also Supplementary Material), there were notable similarities between the two dairy samples and the two PBCA samples. These particularly related to texture where the two PBCA samples were much more ‘firm’ and the two dairy samples were much more ‘light/airy’. For example, citation frequencies for Samples PBCA2 and Dairy1 were, respectively, 76% and 8% (‘firm’) and 6% and 51% (‘light/airy’). A texture difference of smaller magnitude, but nonetheless notable, was ‘creamy/smooth’ mouthfeel, where the citation frequency for Sample Dairy2 was nearly double that of Sample PBCA1 (respectively, 90% and 47%). A similar result was found for ‘dissolves quickly in mouth’ (Dairy1 = 57% and PBCA2 = 24%). Flavour differences were less pronounced, and more specific to individual samples than dictated by product type (PBCA or dairy). For example, ‘buttery’ flavour was most characteristic of Sample Dairy2 (64%) and had a higher citation frequency than found in the other three samples (33–40%). Sample PBCA2 was also the most ‘sweet’, while Sample Dairy1 was the least ‘sweet’ (22% vs. 6%). Sample Dairy1 had the highest citation frequency for ‘sour/tangy’ (51%). ‘Shiny/glossy’ appearance was low in all samples (28% or less), but lowest in Sample PBCA2 (8%). Sample PBCA1 was perceived as considerably more ‘mild/bland’ (46%) than the other three samples (25–32%).

#### 3.2.2. Emotional

Sample separation based on emotional associations (Figure 2B) resembled that from the sensory data, in that the primary difference was related to product type (PBCA or dairy) and was valence-driven. Sample separation based on ‘happy/satisfied’ aligned with the results based on the liking data (Table 1), while ‘unhappy/dissatisfied’ indicated greater differentiation between the four samples, with, for example, Sample PBCA1 being less frequently associated with ‘enthusiastic/inspired’ and more frequently associated with ‘dull/bored’ than the dairy samples. Sample PBCA2 was more frequently associated with ‘blue/uninspired’, ‘tense/bothered’, and ‘jittery/nervous’ than the dairy samples.

#### 3.2.3. Conceptual

The conceptual data primarily separated the samples by product type—PBCA vs. dairy (Figure 2C). Compared to the dairy samples, the PBCA samples were perceived as being more ‘unfamiliar’, ‘artificial’, and ‘cheap’. They were also perceived as being less ‘traditional’, ‘genuine’, and ‘versatile’. The most polarised conceptualisations were seen for Samples PBCA2 and Dairy2, with the former being less ‘comforting’, ‘wholesome’, ‘simple’, and ‘natural’. Samples PBCA1 and Dairy2 were significantly different for ‘nutritious’ (respectively, 12% vs. 24%).

#### 3.2.4. Situational

Aligning with the findings from the three earlier sets of product characterisations, perceived appropriateness for situational uses largely differentiated samples based on product type (PBCA or dairy) (Figure 2D). Compared to the dairy samples, PBCA samples were perceived as less appropriate ‘to have with salmon’, ‘to spread on a cracker with toppings’, ‘as a spread on a bagel/sandwich’, and ‘to dip raw vegetables in’. Minor variations on this theme were observed for the other situational uses, and across the 15 use situations, the average citation frequency for dairy samples was 47–48% compared to 32–33% for PBCA samples. In a few instances, the samples were differently differentiated. Sample Dairy1 was more appropriate ‘to use as an ingredient in a dip/sauce’ than Sample Dairy2 (66% vs. 50%), and both were more appropriate for this use than the PBCA samples (31–32%). Samples PBCA2 and Dairy2 were equally appropriate ‘to use in baking’, and Sample Dairy2 was more appropriate than the other three samples ‘to use as a butter replacement’ (50% vs. 31–36%).

### 3.3. Sensory and Non-Sensory Drivers of Liking (Objective 2)

The results after penalty/lift analysis performed using all participants (n = 157) are shown in Table 3.

#### 3.3.1. Sensory Drivers of Liking

For sensory characteristics, the largest positive impact (hedonic lift) was found for ‘creamy/smooth mouthfeel’ (1.7), followed by ‘dissolves quickly in mouth’ (1.1) and ‘sweet’ (1.1). The mean impact was greater than or equal to 0.5 hedonic scale point for ‘buttery flavour’ (0.9), ‘light/airy texture’ (0.8), ‘mild/bland flavour’ (−0.7), ‘savoury flavour’ (0.6), ‘firm’ (−0.6), and ‘shiny/glossy appearance’ (0.5).

#### 3.3.2. Emotional Drivers of Liking

For emotional product associations, the mean impacts were mostly greater than for sensory characteristics, and with one exception (‘passive/quiet’), these exceeded one hedonic scale point. Specifically, hedonic lifts were established for all emotions with positive valence and for ‘active/alert’, and among these the largest mean impact was observed for ‘happy/satisfied’ (2.1). Hedonic penalties were established for all emotions with negative valence and the largest mean impact was observed for ‘unhappy/dissatisfied’ (−2.7). In absolute values, the hedonic penalties were larger than the hedonic lifts (−1.5 to −2.7 vs. 1.0 to 2.1).

#### 3.3.3. Conceptual Drivers of Liking

Significance tests for mean impact were performed for conceptual terms with average citation frequency greater than 10%, and of these, a hedonic penalty was established for ‘unfamiliar’ (−2.0), ‘cheap’ (−1.8) and ‘artificial’ (−1.6). The hedonic lifts ranged between 0.5 (‘simple’) and 1.8 (‘comforting’), with mean impacts at one hedonic scale point or above also observed for ‘trustworthy’ (1.5), ‘genuine’ (1.5), ‘wholesome’ (1.3), ‘natural’ (1.3) and ‘nutritious’ (1.0). All of these had positive connotations.

#### 3.3.4. Situational Drivers of Liking

Regarding situational use characteristics, significant hedonic lifts were established for all 12 uses, with values from 0.5 (‘to use in baking’) to 1.7 (‘to spread on a cracker with toppings’). The four other use situations with a hedonic lift that was at least one scale point were ‘as a spread on a bagel/sandwich’ (1.6), ‘to have with salmon’ (1.5), ‘to dip raw vegetables in (e.g., carrot, cucumber)’ (1.3), and ‘for sushi filling’ (1.1).

### 3.4. Sensory Drivers of Non-Sensory Product Characteristics (Objective 3)

The sensory drivers of non-sensory product associations were elucidated through logistic regression. Table 4 shows the results based on the analysis including all 157 participants. When the selection of a sensory CATA term increased the odds of a non-sensory CATA term being selected, the OR values ranged between 1.70 and 7.35. Conversely, when the selection of a sensory CATA term decreased the odds of a non-sensory CATA term being selected, the OR values ranged between 0.34 and 0.59.

#### 3.4.1. Appearance, Taste, and Flavour

Selection of ‘shiny/glossy appearance’ was associated with increased citation probability for ‘happy/satisfied’, ‘active/alert’, ‘sophisticated’, and ‘versatile’. In other words, this sensory appearance characteristic was positive in the eyes of consumers.

For taste attributes, the selection of ‘salty’ increased odds of selecting ‘artificial’. For ‘sour/tangy’, there was an increase in selection probability of the emotional associations ‘active/artificial’ and ‘unhappy/dissatisfied’, as well as the conceptual associations ‘artisanal’ and ‘versatile’. However, ‘sour/tangy’ perception was associated with a reduction in the odds of selecting ‘to use in baking’ and ‘to use as butter replacement’. For ‘sweet’, only a single significant relationship was established and pertained to a reduction in the odds of selecting ‘simple’ as a conceptual sample descriptor.

Regarding the cream cheeses’ flavour characteristics, ‘buttery flavour’ increased the odds of selecting ‘happy/satisfied’, ‘comforting’ and ‘to use as a butter replacement’. When ‘cow-like/barnyard flavour’ was selected, the odds of term selection increased for ‘secure/at ease’ and ‘wholesome’. ‘Mild/bland flavour’ had a major impact on increasing selection probability for ‘dull/bored’ (OR = 7.35), with lesser impacts on other deactivated emotional associations (‘relaxed/calm’ and ‘passive/quiet’). There was also an increased probability of selecting ‘simple’ and ‘cheap’ while the selection probability decreased for ‘to dip raw vegetables in’. There was an increased selection probability for emotional associations with opposing valence—‘happy/satisfied’ and ‘jittery/nervous’ when ‘savoury flavour’ was perceived.

#### 3.4.2. Texture and Mouthfeel

For texture characteristics, ‘firm’ was associated with an increased selection probability for ‘tense/bothered’, ‘unfamiliar’, ‘artificial’ and ‘to use as a pizza topping’. However, cream cheeses perceived as ‘firm’ were less likely to be associated with ‘to dip raw vegetables in’ and ‘to spread on a cracker with toppings’. A ‘light/airy texture’ decreased selection probability for ‘unfamiliar’ and increased selection probability for ‘to have with salmon’.

‘Creamy/smooth mouthfeel’ was the sensory attribute for which most significant associations to non-sensory terms were established. The probability of selection increased for positive and/or deactivated emotions (‘happy/satisfied’, ‘secure/at ease’, relaxed/calm’). Similar associations were observed for ‘comforting’, ‘versatile’ and ‘trustworthy’ conceptualisations. Especially large OR values were found for ‘happy/satisfied’ (OR = 7.17) and ‘comforting’ (OR = 4.96). The probability of selecting several use situations also increased (‘to dip raw vegetables in’, ‘as a spread on a bagel/sandwich’, ‘to have with salmon’, ‘to use as an ingredient in a dip/sauce’, ‘in a cheesecake filling’). For ‘dissolves quickly in mouth’ increased selection probability was established for ‘happy/satisfied’, ‘nutritious’, ‘genuine’, ‘versatile’, ‘to dip raw vegetables in’, and ‘as a spread on a bagel/sandwich’.

### 3.5. Consumer Segmentation Based on Liking (Objective 4)

Two groups of participants with different patterns of liking for the PBCA and dairy cream cheeses were established through cluster analysis (Table 2). Of the 152 participants who were entered into this analysis, the majority of participants (n = 111, 73%) liked PBCA and dairy samples equally (all samples between 6.5 and 6.7) (*p* > 0.53), and there was no significant difference between mean scores based on Tukey’s test. However, a minority of participants (n = 41, 27%) liked the two cream cheese samples from dairy (7.1–7.3) considerably more than the two PBCA samples (3.3–5.1) (*p* < 0.001). Correspondingly, the two clusters were named, respectively, *PBCA Likers* and *PBCA Dislikers*.

#### 3.5.1. Product Characterisation by Cluster

**Figure 3:**
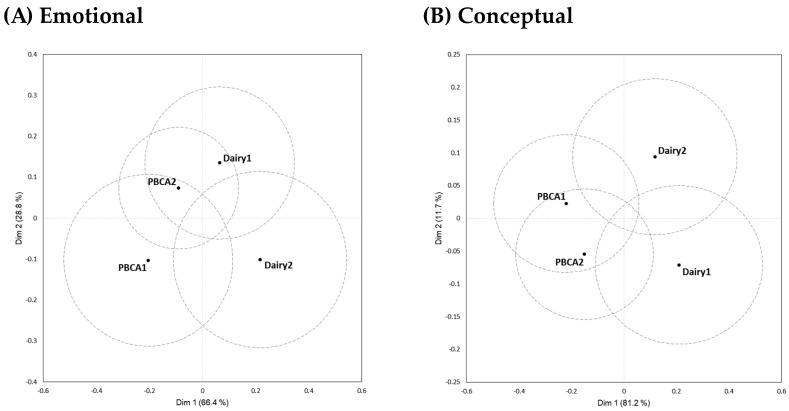

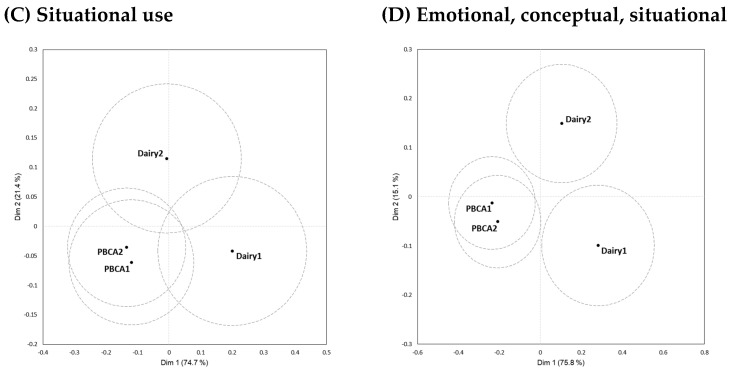
Plots of the first two dimensions after Correspondence Analysis based on data from participants in the *PBCA Likers* cluster (n = 111) showing average samples positions with 95% confidence intervals.

In Table 3, the product characterisation results obtained in the *PBCA Likers* cluster (n = 111) were shown next to those for the total sample (n = 157) and facilitated a comparison of the two sets of results. Across all samples, average term citation frequencies were similar, never differing more than 5% for individual CATA terms. Despite establishing no significant differences in average liking scores in the *PBCA Likers* cluster (Table 2), the four samples of cream cheese were significantly differentiated on 9 of 12 sensory descriptors (excluding ‘cow-like/barnyard’, ‘mild/bland’, ‘savoury flavour’), 3 of 12 emotional product associations (‘happy/satisfied’, ‘unhappy/dissatisfied’, ‘enthusiastic/inspired’), on 5 of 14 conceptual descriptors (‘traditional’, ‘unfamiliar’, ‘versatile’, ‘genuine’, ‘artificial’), and 9 of 12 situational uses (‘to have with salmon’, ‘to spread on a cracker with toppings,‘ ‘as a spread on a bagel/sandwich’, ‘to use as an ingredient in a dip/sauce’, ‘to dip raw vegetables in’, ‘to make icing for cakes’, ‘to use in baking’, ‘to use as a butter replacement’, ‘for a creamy smoothie’). The overlapping 95% confidence sample ellipses in Figure 3A–C reflected these findings by showing that the samples were neither completely similarly nor completely uniformly perceived but were partly different to each other (the exception was the sensory data where sample ellipses were non-overlapping, Supplementary Material). When a joint analysis of all 50 non-sensory terms was performed, a separation of samples was observed (Figure 3D), suggesting that these data contributed insights that extended the non-significant discrimination based on liking, primarily for the two dairy cream cheese samples.

#### 3.5.2. Drivers of Liking by Cluster

The information in Table 3 also showed that the sensory drivers of liking in the total sample and the *PBCA Likers* cluster were largely similar. Notably, there were no product characterisation terms where significant mean impacts in the two sets of results were, respectively, positive and negative (or vice versa). There were, however, multiple instances where the magnitude of the mean impacts differed, reaching 0.5 or 0.6 hedonic scale points, always with the larger value (absolute numbers) in the *PBCA Likers* cluster (‘creamy/smooth mouthfeel’ (1.2 vs. 1.7), ‘unhappy/dissatisfied’ (−2.2 vs. −2.7), ‘tense/bothered’ (−1.3 vs. −1.9), ‘jittery/nervous’ (−1.3 vs. −1.8), ‘cheap’ (−1.2 vs. −1.8), ‘artificial’ (−1.1 vs. −1.6), and ‘as a spread on a bagel/sandwich’ (1.0 vs. 1.6)).

Penalty/lift analysis was also performed in the *PBCA Dislikers* cluster (n = 41). While the results were regarded with considerable caution because of the low sample size, they were, nonetheless, helpful in understanding what sensory characteristics of the PBCA samples were disliked, particularly in the case of Sample PBCA2. The results aligned with those in the total sample (n = 157), but there were multiple terms where the difference in mean impact was one hedonic scale point or more, and always with the more extreme value in the *PBCA Dislikers* cluster. These were: ‘firm’ (−1.9), ‘creamy/smooth mouthfeel’ (2.4), ‘light/airy texture’ (1.9), ‘tense/bothered’ (−2.5), ‘active/alert’ (1.8), ‘unfamiliar’ (−2.5), ‘trustworthy’ (2.3), ‘genuine’ (2.3), ‘artificial’ (−2.5), ‘as a spread on a bagel/sandwich’ (2.5), ‘to dip raw vegetables in’ (2.1), and ‘to use as a pizza topping’ (1.6) (Supplementary Material has full details).

## 4. Discussion

### 4.1. Liking for PB Cream Cheese Alternatives and Consumer Segmentation

One important finding of the present research was that PBCA samples were more likely to be liked than disliked by the 157 participants, with average liking scores close to the verbal anchor ‘like slightly’ (i.e., six on the nine-point hedonic scale). This finding, which links to Objective 1, adds nuance to the narrative that PB alternative foods struggle with poor sensory quality and consumer acceptance [11,18,26,75,76,77]. That is, amid evidence that consumer acceptability will likely be modest or less, there is value in drawing attention to those situations when liking scores for one or more PB alternatives are the same or very similar to those obtained for their non-PB counterparts. One recent example was a US consumer study with burger patties [78]. Under blinded tasting conditions, preferences for the beef burger and the hybrid and pea protein burgers were not significantly different. The present research is another example, and shows, although only in the context of four samples, that PB alternative cream cheese is “within reach” of dairy cream cheese, trailing less than one hedonic scale point (Table 2) when all participants were considered.

Consumer segmentation based on liking was expected and found (Objective 4). The results suggested the existence of two clusters (*PBCA Likers* and *PBCA Dislikers*), although this finding must be regarded with some caution due to low sample sizes. However, since the smaller cluster comprised 41 participants, which aligned with an estimate from Moskowitz [74] that stable means in sensory and consumer research are common based on 40–50 people, it was reasonable to interpret, with care, the results. It resonated with previous studies that a cluster of people were found who disliked PB alternatives [5,79], but the present results were unique in the sense that *PBCA Likers* was the larger cluster and *PBCA Dislikers* was the smaller cluster (73% vs. 27%). The findings of Cardello, Llobell, Giacalone, Roigard, and Jaeger [28] (PB milk alternatives, 345 participants) showed that more fine-grained segmentation is possible, allowing groups of consumers to be identified with varying preferences for different PB product variants. Tentatively, such extended segmentation on a larger consumer sample could have resulted in further differentiation between the two PBCA samples, for example, with one segment disliking both Samples PBCA1 and PBCA2, and another segment that disliked Sample PBCA2 but liked Sample PBCA1 as much as the dairy samples.

The consumer population further contextualised the liking results. Participants in this study stated an interest in reducing their dietary reliance on meat and dairy foods but were not vegetarian or vegan, and they mostly lacked previous experience with PBCA (66% had not tasted PBCA before; Table 1). Higher liking among vegans than omnivores and flexitarians for a range of PB alternative foods was reported by Pointke, Ohlau, Risius, and Pawelzik [26] and, if replicated, this could lend further support to the conclusion that PB products need not be inferior or poorly accepted. Moreover, it could contribute to greater uptake of PB cream cheese alternatives, which in turn, could support the notion of a gateway effect, first to other types of PBCA, then to other types of PB food alternatives, and eventually a more complete transition to a PB diet.

### 4.2. Suggestions for How to Improve PB Cream Cheese Alternatives

The findings linked to Objectives 2 and 3 provided specific insight regarding formulation changes that could be made to improve PB cream cheese alternatives and close the gap to dairy cream cheese in terms of average liking (Table 2).

#### 4.2.1. Texture and Mouthfeel

Most important would be to improve the texture and mouthfeel characteristics of the PBCA samples to make them more similar to the dairy cream cheese variants (Figure 2, Table 3). Because flavour is often mentioned as a driver of disliking for PB alternatives to milk and yoghurt, for example, soy/bean and off-flavour [18,75], the finding that texture and mouthfeel characteristics in the present research were stronger drivers of liking than other sensory modalities was a pertinent reminder of the importance of texture and mouthfeel for consumer acceptance. Muñoz and Civille [80] were among the first to draw attention to this, and among PB meat alternatives, there are many reports that poor texture is key to consumer dislike [81]. Considering also that texture and mouthfeel are very important to consumer acceptability for dairy cheeses of different types [23,35], including cream cheese [22], establishing these sensory modalities as very important for PB cream cheese alternatives, therefore, had face validity.

The present results predicted that PBCA samples would be more liked if the texture was less firm and more light/airy, the mouthfeel was more smooth/creamy, and the cheese dissolved more quickly in mouth (Table 3). A comparison of the results for the *PBCA Likers* cluster against the indicative results for the *PBCA Dislikers* cluster suggested that firmness, conversely, lack of light/airy texture, was the major culprit, followed by insufficient smooth/creamy mouthfeel. For these sensory descriptors, the biggest differences in the mean impact on liking were established (1.2–1.7 scale points) (Table 3, Supplementary Material). Based on logistic regression (Table 4), it was seen that samples perceived as ‘firm’ increased the odds of selecting ‘tense/bothered’, ‘unfamiliar’, and ‘artificial’ with OR values ranging between 2.13 and 3.21 (i.e., more than 200% increase in terms of selection probability). The PBCA samples were so firm that they were not easily spreadable, and this could be what primarily evoked the associations with ‘unfamiliar’ since dairy cream cheeses are much softer (Figure 2). The findings for ‘light/airy texture’ supported this interpretation as selection of this sensory term majorly decreased the probability of selecting ‘unfamiliar’ (OR = 0.34). Firm texture was also linked to reduced uses—‘to dip raw vegetable in’ (OR = 0.46) and ‘to spread on a cracker’ (OR = 0.57)—which, in turn, could have contributed to evoking increased feelings of ‘tense/bothered’ because ways that cream cheese is commonly used were no longer regarded as appropriate. The finding that firm cream cheese was regarded more appropriate as a pizza topping could tentatively be harnessed for vegan pizza, which is gaining market share [82].

When cream cheese was perceived as having a ‘creamy/smooth mouthfeel’, a very large increase in selection probability for ‘happy/satisfied’ was observed (OR = 7.17) and emphasised the importance of this sensory characteristic for consumer acceptability. When further considering the linkages to emotional and conceptual product characterisations, it seemed that low firmness in combination with a smooth and creamy mouthfeel should be pursued. This was supported by increased selection probability for positive and deactivated emotions (‘secure/at ease’, ‘relaxed/calm’) and for ‘comforting’ and ‘trustworthy’, where the latter stood in opposition to ‘unfamiliar’ and ‘artificial’. Increased versatility was also inferred (OR = 3.32), and this was further reflected in increased odds for five situational uses.

In terms of consumer uptake for PB cream cheese alternatives, the collective results for texture and mouthfeel characteristics indicate that if the focal sensory properties can be improved, it will not only positively impact liking, but also reduce the multiple barriers that consumers experience in switching to PB alternative foods [47]. Additional positive/desirable conceptualisations will be a benefit, and in this regard, one result for ‘dissolves quickly in mouth’ was somewhat intriguing. While the results largely resembled those for ‘creamy/smooth mouthfeel’, an exception was ‘nutritious’, for which selection probability was also significantly increased (OR = 2.72). A possible explanation for this linkage is that samples with a quickly disappearing mouthfeel feel less greasy/oily, which, in turn, may make them appear healthier. This may feed into the health halo that is associated with PB alternative foods, which in many instances, is not supported based on nutritional content for individual products in the dairy category [83], although there is supporting evidence for PB diets overall [2]. In the present case, it may, therefore, be necessary to identify a positive nutritional link to ‘dissolves quickly in the mouth’ if this conceptualisation is to be harnessed for marketing purposes.

#### 4.2.2. Appearance, Taste, and Flavour

Texture aside, ‘sweet’ taste exerted that largest influence on the mean liking for the tested cream cheeses (Table 3). That the impact was a hedonic lift (1.1) fitted expectations, since sweet liking is an innate preference in humans [84]. However, it is important to be mindful that sweet liking determined in a test setting may not translate when products are consumed in larger quantities and across longer periods of time [85]. With this caution in mind, it was interesting to note that only one significant association between ‘sweet’ and non-sensory characteristics was established (Table 4). Tentatively, it could indicate that increased sweetness will increase immediate liking, but lack effectiveness in strengthening the emotional, conceptual, and situational uses, and hereby not significantly help to overcome the multi-dimensional barriers that that many consumers face when seeking to eat a more PB-rich diet [47].

A tangible example of the value of the multi-response measurement approach was seen for ‘sour/tangy’, which, according to the penalty/lift analysis, did not significantly impact mean liking (Table 3). Yet, selection of this term increased the odds of selecting ‘unhappy/dissatisfied’, which made sense in the context of ‘sweet’ increasing selection probability for ‘happy/satisfied’. However, the result for ‘sour/tangy’ was more complex due to this characteristic also increasing the odds of a sample being associated with ‘active/alert’, ‘artisanal’, and ‘versatile’. The latter, nonetheless, excluded certain uses (‘to use in baking’ and ‘to use as butter replacement’) (Table 4), and possibly ‘versatile’ needed to be interpreted in the context of ‘artisanal’ and be seen as indicative of more sophisticated uses. Sample Dairy1, which was the most ‘sour/tangy’, was most strongly associated with ‘to have with salmon’. Considering the results for ‘sweet’ and ‘sour/tangy’ collectively, these sensory characteristics must be balanced for high consumer acceptance. Fruit-based products demonstrate this, for example, oranges [86], apple juice [87], and champagne [88].

There were few specific results for ‘salty’ other than increasing the selection odds for ‘artificial’. Despite not exerting a significant impact in the penalty/lift analysis (Table 3), this result nonetheless indicated that caution is warranted since ‘artificial’ has negative connotations. The actual salt content was higher in the two PBCA samples than in the dairy samples (855–979 mg/100 g vs. 350–411 mg/100 g) (Supplementary Material), and Sample PBCA2 was perceived as the saltiest. Sample PBCA2 was also the least liked by a majority of participants (i.e., in the *PBCA Dislikers* cluster) (Table 2). Salt content in PB alternative foods, including dairy, has been identified as a possible concern [83], so a way to reduce it should perhaps be sought, both as a small contribution toward reducing salt intake, which is much above the recommendations in many countries [89], and because it may contribute negatively to consumers’ product experience.

Cream cheese samples (PBCA and dairy) perceived as having a ‘mild/bland flavour’ were less liked according to the penalty/lift analysis (Table 3). This finding provided further evidence of a relationship that seems to be generic rather than product specific. For product innovation, this insight does not imply that strong flavour is preferred since such foods are generally also disliked. Instead, the challenge seems to be to find a balance where products are perceived as neither one nor the other, and additionally avoid specific flavours and tastes that penalise liking while promoting those that exert a hedonic lift. For cream cheese, one of the flavour attributes that exerted a hedonic lift was ‘buttery flavour’ (Table 4). This flavour characteristic also increased the likelihood of regarding a cream cheese sample as suitable ‘to use as a butter replacement’. Considering that the PBCA samples contained coconut oil as a major ingredient (Supplementary Material), it is not immediately clear how this flavour characteristic can be naturally introduced, but if it could, PBCA may become more appealing to people who watch their cholesterol intake and so limit their intake of butter.

‘Shiny/glossy appearance’ was not strongly characteristic of any of the four samples but applied significantly more to the dairy than PBCA samples. As a target for product innovation, smaller rather than larger changes would, thus, be sought and the possible benefit is a positive impact on the mean liking, as well as increased selection probability for ‘happy/satisfied’, ‘active/alert’, ‘sophisticated’, and ‘versatile’. Mindful of the results for ‘sour/tangy’ regarding versatility, it may be prudent to explore further how ‘shiny/glossy appearance’ is linked to perceived versatility. Qualitative research methods [90] may be suitable, working with end consumers, chefs, and others in the catering business.

### 4.3. Non-Sensory Product Characterisation

The results for the *PBCA Likers* cluster (n = 111) showed that samples, despite not being significantly different in terms of the degree of liking/disliking (Table 2), differed on several non-sensory product perceptions, especially situational appropriateness (Table 3). This result captured the essence of why appropriateness of use is part of the “beyond liking” paradigm that has gained acceptance in sensory and consumer research. Moreover, this result illustrated with clarity that samples that are not differently liked can be regarded as differently appropriate in various use situations, and this is what originally motivated Cardello and Schutz [43] to advocate for the inclusion of situational appropriateness in consumer-centric product research.

Qualitative research has already been suggested as a useful path for further consumer research with cream cheese [90], and online product reviews may be a data source option [91]. In particular, it could be interesting to take situational uses and perceived appropriateness as a starting point for identifying sensory innovation targets. The present results suggested, for example, that firmness was key in samples being regarded as more vs. less appropriate for certain uses. This characteristic may be more important than its ingredients (PB or dairy), and samples that systematically vary in firmness (and related texture characteristics such as ‘spreadability’ and ‘light/airy’) could be studied. The results linked to the use of cream cheese in sushi rolls are also interesting because of its popularity despite not representing traditional sushi. New uses for cream cheese may be uncovered, possibly in markets where this product is not well established, which can guide the way for PB alternative cream cheese with different taste and texture profiles that possibly also draw on the long history of soy-based cream cheese [59] and take advantage of its lactose-free status for the benefit of Asian populations where lactose intolerance is more prevalent [92]. More generally, a focus on situational use connects with the literature around being a mindful host, which includes serving PB and vegetarian meals [93].

### 4.4. Limitations and Suggestions for Future Research

All empirical studies have limitations, and this is no exception. The inclusion of additional research participants to confirm the smaller *PBCA Dislikers* cluster is needed. A replication and extension research strategy would be appropriate, and this would ideally include a larger number of cream cheese samples—dairy and PBCA. If the latter were made from ingredients other than coconut oil, this would be interesting, for example, cashew nuts, oats, almonds, and soy [63]. At the time of the study, such products were not available in major retail outlets in New Zealand, but they do exist in other countries, and some have higher protein content than the PBCA samples in this research.

Care is needed in generalising the results to other consumer nationalities. For example, Foguel, Neves Rodrigues Ract, and Claro da Silva [22] found that the most liked sample of cream cheese (dairy) in their study with US consumers was more firm and less spreadable. This differs from the present result, but may also reflect greater variation in cream cheese on the US market, where, for example, the popular Philadelphia brand sells cream cheese in many flavours, several of which are available in different textures that vary in firmness and spreadability [58]. For applied and marketing purposes, it is advisable that segmentation be refined beyond consumer nationality, for example, by considering key demographic variables such as age and gender.

Only about one third of participants had previous experience with PBCA (Table 1), and this limited the value of a comparison of responses between those who did and did not have familiarity. However, it could be relevant to purposefully recruit participants with PBCA experience to uncover their sensory and non-sensory product perceptions and better understand what role familiarity plays in acceptance of PBCA.

The “beyond liking” paradigm of sensory and consumer science implemented in the present research demonstrated that sample differentiation based on non-sensory terms can be achieved when not observed on liking data alone (Figure 3D). Similar findings have been observed in other product categories [28]. For product developers, it adds additional direction for innovation efforts, but ways to reduce ballot length would be welcome. Additional analyses of the present data could identify a subset of non-sensory attributes that most effectively deliver additional sample differentiation. Over time, similar analyses could be performed for other studies and contribute to guidance regarding the most important non-sensory aspects to include. Emotional activation is likely to be among them, but it is less clear what criteria can be used to reduce the number of conceptual and situational use terms. Empirical as well as theoretical advances are needed.

## 5. Conclusions

In a case study approach, with cream cheese (commercially available) as the product category, the present research presented an in-depth consumer study that compared four samples, of which two were PBCA and two were dairy (from cow’s milk). Regarding liking, the key finding was that the majority of participants (i.e., *PBCA Likers* cluster) gave mean liking scores to the four samples that were not statistically different. This was a positive result for PB alternative foods, which often struggle against a narrative of inferior sensory quality and unmet consumer expectations. Penalty/lift analysis determined drivers of liking (sensory and non-sensory), and these offer tangible guidance for innovation efforts and may have relevance for the wider PBCA category. Situational appropriateness data suggested that PB cream cheese alternatives that were firmer than dairy cream cheese had less applicability than dairy cream cheese for some uses. The main limitation of the work lay in the use of a small number of samples and that the PBCA samples were made from coconut only. Ample scope exists to extend the research and confirm its generalisability. Nonetheless, the key takeaway from this research was that PBCA have the potential to match their dairy counterparts in the eyes of consumers. Amidst a dominant narrative of the opposite, the results are encouraging for the transition to dietary habits that are less dependent on meat and dairy.

## Figures and Tables

**Table 1:** Participant characteristics (%) ^$^, for the total sample (*n* = 157) and the consumer segment where cream cheese liking for plant-based cheese alternatives (PBCA) was as high as liking for dairy cheeses (*PBCA Likers*, *n* = 111).

Participant Characteristics	Total Sample *n* = 157	PBCA Likers *n* = 111
**Gender**		
Male	50	51
Female	50	49
**Age Group**		
Younger (18–39 y.o.)	50	55
Older (40–66 y.o.)	50	45
Ethnicity *		
New Zealand European	54	53
Māori	7	5
Pacific Island	6	8
European	4	4
North American (Canada and US)	1	0
Chinese	11	14
Indian	15	15
Southeast Asian	11	9
African and Middle Eastern	4	4
Household composition *		
No one, I live alone	11	13
Spouse/partner	59	58
Children aged under 18	35	33
Children aged over 18	17	18
Parents	17	18
Flatmate/s	10	11
Other **	4	5
**Number of people in household**		
1–2 people	40	39
3–4 people	47	50
5 or more people	13	12
**Household income (NZD per year)**		
Less than NZD 50,000	6	5
NZD 50,000–NZD 79,999	13	7
NZD 80,000–NZD 99,999	10	11
NZD 100,000–NZD 119,999	18	20
NZD 120,000–NZD 149,999	13	13
NZD 150,000 or more	32	36
I’d prefer not to say	7	8
**Previous plant-based/dairy-free cheese consumption**		
Yes	34	35
No	66	65
Cream cheese—stated liking ***	7.7 ± 1.3	7.6 ± 1.3
**Cream cheese—stated consumption frequency**		
Every day or almost everyday	2	2
2 or more times a week	13	12
About once a week	16	18
About 2–3 times a month	27	25
About once a month	19	18
About once every 2–3 months	18	20
About once a year or less	3	5
**Cream cheese—stated purchase frequency**		
Have purchased in the last 2 months	58	58
Have purchased in the last 3–12 months	38	39
Have never purchased	4	4

Notes: ^$^ After rounding, some percentage values do not add to 100. * Total responses may exceed 100% because multiple options can be selected. ** Other included extended family, and siblings. *** Stated liking obtained on a 9-pt fully labelled scale with the anchors ‘Dislike extremely’ (1), ‘Dislike very much’ (2), ‘Dislike moderately’ (3), ‘Dislike slightly’ (4), ‘Neither like nor dislike’ (5), ‘Like slightly’ (6), ‘Like moderately’ (7), ‘Like very much’ (8), and ‘Like extremely’ (9), where the shown values are expressed as mean ± standard deviation around the mean.

**Table 2:** Cream cheese samples (plant-based cheese alternatives (PBCA) and dairy (Dairy)) used in the research, showing mean liking scores (‘dislike extremely’ = 1 and ‘like extremely’ = 9) with standard deviations shown in brackets and Tukey’s HSD *post hoc* comparison of means shown in superscript ^#^. Values shown for total sample and the two retained preference clusters ^##^.

Sample	Mean Liking *Total Sample* n = 157	Mean Liking *PBCA Likers* n = 111	Mean Liking *PBCA Dislikers* n = 41
PBCA1	6.1 ^b^ (1.7)	6.5 ^a^ (1.5)	5.1 ^b^ (1.8)
PBCA2	5.9 ^b^ (2.0)	6.7 ^a^ (1.4)	3.3 ^c^ (1.1)
Dairy1	6.8 ^a^ (1.6)	6.6 ^a^ (1.7)	7.3 ^a^ (1.1)
Dairy2	6.8 ^a^ (1.5)	6.7 ^a^ (1.7)	7.1 ^a^ (1.1)

Notes: ^#^ Tukey’s HSD was used for sample mean comparisons. Within columns, samples with different letters had significantly different means at the 5% level of significance. ^##^ Five participants were excluded prior to cluster analysis.

**Table 3:** Drivers of liking for cream cheese (plant-based cheese alternatives (PBCA) and dairy). Results for sensory and non-sensory terms (rows) for total sample (n = 157) and *PBCA Likers* cluster (n = 111). Within sample grouping (sets of 3 columns), Column 1 contains *p*-values from Cochran’s Q test for sample differences, Column 2 contains average term citation frequencies (%) across all samples in the study, and Column 3 contains the mean impact on liking ^$^ when the CATA term was selected.

Response Type and Term	Total Sample *p*-Value from Cochran’s Q Test	Total Sample Average Citation Frequency (%)	Total Sample Mean Impact on Liking ^$$,$$$^	PBCA Likers *p*-Value from Cochran’s Q Test	PBCA Likers Average Citation Frequency (%)	PBCA Likers Mean Impact on Liking ^$$,$$$^
**Sensory**						
Shiny/glossy appearance	<0.0001	18.6	0.5 **	0.000	18.7	0.2 ^ns^
Cow-like/barnyard flavour	0.285	14.3	0.1 ^ns^	0.108	14.9	−0.1 ^ns^
Buttery flavour	<0.0001	43.3	0.9 ***	<0.0001	45.9	0.7 ***
Mild/bland flavour	0.0001	32.3	−0.7 ***	0.064	31.8	−0.6 ***
Salty	<0.0001	17.2	0.0 ^ns^	0.002	15.8	0.2 ^ns^
Savoury flavour	0.165	32.8	0.6 ***	0.821	32.7	0.5 ***
Firm	<0.0001	44.9	−0.6 ***	<0.0001	43.7	−0.2 ^ns^
Sweet	<0.0001	13.7	1.1 ***	0.016	14.2	1.0 ***
Sour/tangy	<0.0001	36.1	−0.2 ^ns^	<0.0001	38.5	−0.1 ^ns^
Creamy/smooth mouthfeel	<0.0001	66.9	1.7 ***	<0.0001	70.9	1.2 ***
Dissolves quickly in mouth	<0.0001	38.4	1.1 ***	<0.0001	42.3	0.9 ***
Light/airy texture	<0.0001	24.4	0.8 ***	<0.0001	25.9	0.5 **
**Emotional**						
Energetic/Excited	0.281	13.9	1.4 ***	0.567	16.7	1.3 ***
Enthusiastic/Inspired	0.002	15.1	1.5 ***	0.020	17.3	1.3 ***
Happy/Satisfied	<0.0001	41.9	2.1 ***	0.034	43.2	1.9 ***
Secure/At ease	0.009	28.8	1.3 ***	0.378	30.0	1.1 ***
Relaxed/Calm	0.001	40.3	1.4 ***	0.300	41.9	1.1 ***
Passive/Quiet	0.003	24.4	−0.1 ^ns^	0.163	26.4	−0.4 *
Dull/Bored	0.003	18.9	−1.5 ***	0.343	18.2	−1.3 ***
Blue/Uninspired	0.000	13.2	−1.9 ***	0.314	11.5	−1.5 ***
Unhappy/Dissatisfied	<0.0001	15.1	−2.7 ***	0.142	12.4	−2.2 ***
Tense/Bothered	<0.0001	11.1	−1.9 ***	0.067	9.7	−1.3 ^n/a^
Jittery/Nervous	0.003	5.3	−1.8 ^n/a^	0.212	4.5	−1.3 ^n/a^
Active/Alert	0.463	17.0	1.0 ***	0.423	19.1	0.8 ***
**Conceptual**						
Comforting	<0.0001	33.3	1.8 ***	0.240	34.5	1.6 ***
Traditional	0.000	30.4	0.9 ***	0.049	31.8	0.5 ***
Cheap	0.000	19.1	−1.8 ***	0.477	16.0	−1.2 ***
Unfamiliar	<0.0001	23.9	−2.0 ***	0.009	20.9	−1.6 ***
Wholesome	0.010	22.3	1.3 ***	0.089	24.1	1.0 ***
Trustworthy	0.038	17.5	1.5 ***	0.739	19.6	1.3 ***
Sophisticated	0.672	11.3	1.1 ***	0.962	13.1	1.0 ***
Simple	0.019	44.1	0.5 ***	0.431	44.4	0.2 ^ns^
Natural	0.001	36.3	1.3 ***	0.259	36.7	1.1 ***
Artisanal	0.626	9.9	0.5 ***	0.449	10.6	0.4 ***
Nutritious	0.004	17.7	1.0 ***	0.087	18.7	0.8 ***
Versatile	<0.0001	24.2	0.8 ***	0.001	24.5	0.6 ***
Genuine	<0.0001	27.7	1.5 ***	0.001	30.0	1.3 ***
Artificial	<0.0001	20.5	−1.6 ***	0.001	19.1	−1.1 ***
**Situational**						
To have with salmon	<0.0001	41.2	1.5 ***	<0.0001	43.0	1.2 ***
To spread on a cracker with toppings	<0.0001	59.2	1.7 ***	0.010	62.4	1.4 ***
As a spread on a bagel/sandwich	<0.0001	64.2	1.6 ***	0.001	68.7	1.0 ***
To use as an ingredient in a dip/sauce	<0.0001	44.7	0.9 ***	<0.0001	45.3	0.7 ***
To dip raw vegetables in (e.g., carrot, cucumber)	<0.0001	40.8	1.3 ***	<0.0001	41.4	1.0 ***
To make icing for cakes	0.000	27.9	0.6 ***	0.000	30.0	0.6 ***
In a cheesecake filling	0.036	42.7	0.9 ***	0.362	44.8	0.7 ***
To use in baking (e.g., muffins)	0.004	40.9	0.5 ***	0.032	41.0	0.6 ***
For sushi filling	0.117	33.1	1.1 ***	0.783	34.7	0.8 ***
To use as a butter replacement	0.001	37.7	0.6 ***	0.001	40.3	0.4 ***
For a creamy smoothie	0.002	19.7	0.7 ***	0.018	20.7	0.7 ***
To use as a pizza topping	0.847	27.5	0.8 ***	0.508	29.5	0.5 ***

Notes: ^$^ 9-point liking response scale spanned between ‘dislike extremely’ (1) and ‘like extremely’ (9). ^$$^ Notation for significance level: *** if *p* < 0.001, ** if *p* < 0.01 and * if *p* < 0.05. Else ^ns^. ^$$$^ Significance testing was not performed when average citation frequency was below 10% (shown as ‘n/a’).

**Table 4:** Results for cream cheese samples (plant-based cheese alternatives (PBCA) and dairy) based on the total sample of 157 participants where CATA terms for sensory product characteristics were significant predictors of non-sensory (emotional, conceptual, and situational) product associations ^#^. Analysis performed using logistic regression, and values in superscript are odds ratios (OR) for non-sensory CATA term selection. Within the emotional, conceptual, and situational categories, non-sensory CATA terms are ordered from most to least impact based on OR values.

Sensory CATA Term	Category of Predicted Non-Sensory CATA Term ^##^	Selection of Sensory CATA Term Increased the Probability (Odds) of Non-Sensory CATA Term Selection	Selection of Sensory CATA Term Decreased the Probability (Odds) of Non-Sensory CATA Term Selection
**Appearance**			
**Shiny/glossy**	Emotional	Happy/satisfied ^2.58^ Active/alert ^2.35^	
	Conceptual	Sophisticated ^2.50^ Versatile ^1.99^	
	Situational		
**Taste**			
**Salty**	Emotional		
	Conceptual	Artificial ^2.40^	
	Situational		
**Sour/tangy**	Emotional	Active/alert ^2.87^ Unhappy/dissatisfied ^2.14^	
	Conceptual	Artisanal ^2.39^ Versatile ^1.70^	
	Situational		To use in baking (e.g., muffins) ^0.54^ To use as a butter replacement ^0.59^
**Sweet**	Emotional		
	Conceptual		Simple ^0.43^
	Situational		
**Flavour**			
**Buttery**	Emotional	Happy/satisfied ^2.27^	
	Conceptual	Comforting ^1.97^	
	Situational	To use as a butter replacement ^2.83^	
**Cow-like/barnyard**	Emotional	Secure/at ease ^2.36^	
	Conceptual	Wholesome ^2.33^	
	Situational		
**Mild/bland**	Emotional	Dull/bored ^7.35^ Relaxed/calm ^2.15^ Passive/quiet ^2.00^	
	Conceptual	Simple ^3.82^ Cheap ^2.22^	
	Situational		To dip raw vegetables in (e.g., carrot, cucumber) ^0.46^
**Savoury**	Emotional	Jittery/nervous ^2.99^ Happy/satisfied ^2.09^	
	Conceptual		
	Situational		
**Texture**			
**Firm**	Emotional	Tense/bothered ^3.21^	
	Conceptual	Unfamiliar ^2.68^ Artificial ^2.13^	
	Situational	To use as a pizza topping ^2.29^	To dip raw vegetables in (e.g., carrot, cucumber) ^0.46^ To spread on a cracker with toppings ^0.57^
**Light/airy**	Emotional		
	Conceptual		Unfamiliar ^0.34^
	Situational	To have with salmon ^2.07^	
**Mouthfeel**			
**Creamy/smooth**	Emotional	Happy/satisfied ^7.17^ Secure/at ease ^2.60^ Relaxed/calm ^2.51^	
	Conceptual	Comforting ^4.96^ Versatile ^3.32^ Trustworthy ^3.28^	
	Situational	To dip raw vegetables in (e.g., carrot, cucumber) ^3.12^ As a spread on a bagel/sandwich ^2.49^ To have with salmon ^2.34^ To use as an ingredient in a dip/sauce ^2.10^ In a cheesecake filling ^1.90^	
**Dissolves quickly**	Emotional	Happy/satisfied ^2.62^	
	Conceptual	Nutritious ^2.72^ Genuine ^2.25^ Versatile ^2.20^	
	Situational	To dip raw vegetables in (e.g., carrot, cucumber) ^2.17^ As a spread on a bagel/sandwich ^2.05^	

Notes: ^#^ Empty cells indicate that no sensory CATA terms were significant predictors (at the 1% level of significance) of non-sensory terms. ^##^ ‘Helps me feel satisfied with life’ and ‘Made using traditional recipes and processes’ were placed in the CATA question with sustainability terms during data collection.

## Data Availability

The original contributions presented in the study are included in the article/supplementary material, further inquiries can be directed to the corresponding author.

## Supplementary Materials

The following supporting information can be downloaded at: https://www.mdpi.com/article/10.3390/foods13040567/s1, Part 1. Supplementary sample information; Part 2. Image of samples as served to participants, in small glass bowl with piece of bread stick. A 3-digit random code was placed on the glass bowl to identify the sample; Part 3. Product characterisations for cream cheese by sensory and non-sensory descriptors based on responses from 157 participants (total sample); Part 4. Plots of the first two dimensions after Correspondence Analysis based on data from total sample (n = 157), showing average sample positions with 95% confidence ellipses; Part 5. Plot of the first two dimensions after Correspondence Analysis based on data from *PBCA Likers* cluster (n = 111), showing average sample positions with 95% confidence ellipses based on sensory descriptors; Part 6. Drivers of liking for cream cheese (PBCA and dairy). Results for sensory and non-sensory terms (rows) in the *PBCA Dislikers* cluster (n = 41).

## References

[1] McClements D.J., Grossmann L. (2024). Next-generation plant-based foods: Challenges and opportunities. Annu. Rev. Food Sci. Technol..

[2] Willett W., Rockström J., Loken B., Springmann M., Lang T., Vermeulen S., Garnett T., Tilman D., DeClerck F., Wood A. (2019). Food in the Anthropocene: The EAT–Lancet Commission on healthy diets from sustainable food systems. Lancet.

[3] World Resources Institute Creating a Sustainable Food Future. https://www.wri.org/research/creating-sustainable-food-future.

[4] Schösler H., Boer J.d., Boersema J.J. (2012). Can we cut out the meat of the dish? Constructing consumer-oriented pathways towards meat substitution. Appetite.

[5] Cardello A.V., Llobell F., Giacalone D., Chheang S.L., Jaeger S.R. (2022). Consumer preference segments for plant-based foods: The role of product category. Foods.

[6] Onwezen M.C., Reinders M.J., Verain M.C.D., Snoek H.M. (2019). The development of a single-item Food Choice Questionnaire. Food Qual. Prefer..

[7] Veganuary Veganuary 2021: The Official Survey Results Are in!. https://veganuary.com/veganuary-2021-survey-results.

[8] Fox P.F., McSweeney P.L.H., McSweeney P.L.H., Fox P.F., Cotter P.D., Everett D.W. (2017). Cheese: An overview. Cheese.

[9] McSweeney P.L.H., Ottogalli G., Fox P.F., Fox P.F., McSweeney P.L.H., Cogan T.M., Guinee T.P. (2004). Diversity of cheese varieties: An overview. Cheese: Chemistry, Physics and Microbiology.

[10] Grossmann L., McClements D.J. (2021). The science of plant-based foods: Approaches to create nutritious and sustainable plant-based cheese analogs. Trends Food Sci. Technol..

[11] Falkeisen A., Gorman M., Knowles S., Barker S., Moss R., McSweeney M.B. (2022). Consumer perception and emotional responses to plant-based cheeses. Food Res. Int..

[12] Grasso N., Roos Y.H., Crowley S.V., Arendt E.K., O’Mahony J.A. (2021). Composition and physicochemical properties of commercial plant-based block-style products as alternatives to cheese. Future Foods.

[13] McClements D.J. (2020). Development of next-generation nutritionally fortified plant-based milk substitutes: Structural design principles. Foods.

[14] Short E.C., Kinchla A.J., Nolden A.A. (2021). Plant-based cheeses: A systematic review of sensory evaluation studies and strategies to increase consumer acceptance. Foods.

[15] Appiani M., Cattaneo C., Laureati M. (2023). Sensory properties and consumer acceptance of plant-based meat, dairy, fish and eggs analogs: A systematic review. Front. Sustain. Food Syst..

[16] Chavan R., Jana A. (2007). Cheese substitutes: An alternative to natural cheese—A review. Int. J. Food Sci, Techol. Nutr..

[17] Jeske S., Zannini E., Arendt E.K. (2018). Past, present and future: The strength of plant-based dairy substitutes based on gluten-free raw materials. Food Res. Int..

[18] Waehrens S.S., Faber I., Gunn L., Buldo P., Bom Frøst M., Perez-Cueto F.J.A. (2023). Consumers’ sensory-based cognitions of currently available and ideal plant-based food alternatives: A survey in Western, Central and Northern Europe. Food Qual. Prefer..

[19] Amyoony J., Moss R., Dabas T., Gorman M., Ritchie C., LeBlanc J., McSweeney M.B. (2023). An investigation into consumer perception of the aftertaste of plant-based dairy alternatives using a word association task. Appl. Food Res..

[20] Drake S.L., Gerard P.D., Drake M.A. (2008). Consumer preferences for mild cheddar cheese flavors. J. Food Sci..

[21] Drake S.l., Lopetcharat K., Clark S., Kwak H.s., Lee S.y., Drake M.a. (2009). Mapping differences in consumer perception of sharp cheddar cheese in the United States. J. Food Sci..

[22] Foguel A., Neves Rodrigues Ract J., Claro da Silva R. (2021). Sensory characterization of commercial cream cheese by the consumer using check-all-that-apply questions. J. Sensory Stud..

[23] Murray J.M., Delahunty C.M. (2000). Consumer preference for Irish farmhouse and factory cheeses. Irish J. Agric. Food Res..

[24] Arise A.K., Opaleke D.O., Salami K.O., Awolola G.V., Akinboro D.F. (2020). Physico-chemical and sensory properties of a cheese-like product from the blend of soymilk and almond milk. Agrosearch.

[25] Oyeyinka A.T., Odukoya J.O., Adebayo Y.S. (2019). Nutritional composition and consumer acceptability of cheese analog from soy and cashew nut milk. J. Food Process. Preserv..

[26] Pointke M., Ohlau M., Risius A., Pawelzik E. (2022). Plant-based only: Investigating consumers’ sensory perception, motivation, and knowledge of different plant-based alternative products on the market. Foods.

[27] Alsado C., Lopez-Aldana L., Chen L., Wismer W. (2023). Consumer perception and sensory drivers of liking of fortified oat milks. Foods.

[28] Cardello A.V., Llobell F., Giacalone D., Roigard C.M., Jaeger S.R. (2022). Plant-based alternatives vs dairy milk: Consumer segments and their sensory, emotional, cognitive and situational use responses to tasted products. Food Qual. Prefer..

[29] Desmet P.M.A., Schifferstein H.N.J. (2008). Sources of positive and negative emotions in food experience. Appetite.

[30] Thomson D.M., Meiselman H.L. (2016). Conceptual profiling. Emotion Measurement.

[31] Grossmann L., Kinchla A.J., Nolden A., McClements D.J. (2021). Standardized methods for testing the quality attributes of plant-based foods: Milk and cream alternatives. Compr. Rev. Food Sci. Food Saf..

[32] Sandine W.E., Elliker P.R. (1970). Microbially induced flavors and fermented foods. Flavor in fermented dairy products. J. Agric. Food Chem..

[33] Meals S.E., Schiano A.N., Drake M.A. (2020). Drivers of liking for Cheddar cheese shreds. J. Dairy Sci..

[34] Ojeda M., Etaio I., Valentin D., Dacremont C., Zannoni M., Tupasela T., Lilleberg L., Pérez-Elortondo F.J. (2021). Effect of consumers’ origin on perceived sensory quality, liking and liking drivers: A cross-cultural study on European cheeses. Food Qual. Prefer..

[35] Shepard L., Miracle R.E., Leksrisompong P., Drake M.A. (2013). Relating sensory and chemical properties of sour cream to consumer acceptance. J. Dairy Sci..

[36] Ciobanu M.-M., Ciobotaru M.-C., Manoliu D.-R., Boișteanu P.-C. (2021). The role of sensory evaluation in food quality control, food research and development: A case of cream cheese study. Sci. Publ. Iași Univ. Life Sci. (IULS).

[37] Sandhya P.S., Lakshmy Priya S. (2017). Formulation of beetroot cream cheese spread. Int. J. Inf. Res. Rev..

[38] Meiselman H.L. (2016). Emotion Measurement.

[39] Prescott J. (2017). Some considerations in the measurement of emotions in sensory and consumer research. Food Qual. Prefer..

[40] Spinelli S., Dinnella C., Ares G., Abbà S., Zoboli G., Monteleone E. (2019). Global Profile: Going beyond liking to better understand product experience. Food Res. Int..

[41] Thomson D.M., Coates T., Meiselman H.L. (2021). Concept profiling–navigating beyond liking. Emotion Measurement.

[42] Cardello A.V., Jaeger S.R., Meiselman H.L. (2016). Measurement of consumer product emotions using questionnaires. Emotion Measurement.

[43] Cardello A.V., Schutz H.G. (1996). Food appropriateness measures as an adjunct to consumer preference/acceptability evaluation. Food Qual. Prefer..

[44] Iacobucci D., Churchill G. (2018). Marketing Research: Methodological Foundations.

[45] Spinelli S., Masi C., Zoboli G.P., Prescott J., Monteleone E. (2015). Emotional responses to branded and unbranded foods. Food Qual. Prefer..

[46] Jaeger S.R., Spinelli S., Ares G., Monteleone E. (2018). Linking product-elicited emotional associations and sensory perceptions through a circumplex model based on valence and arousal: Five consumer studies. Food Res. Int..

[47] Jaeger S.R., Giacalone D. (2021). Barriers to consumption of plant-based beverages: A comparison of product users and non-users on emotional, conceptual, situational, conative and psychographic variables. Food Res. Int..

[48] Cardello A.V., Llobell F., Jin D., Ryan G.S., Jaeger S.R. (2024). Sensory drivers of liking, emotions, conceptual and sustainability concepts in plant-based and dairy yoghurts. Food Qual. Prefer..

[49] Næs T., Varela P., Berget I. (2018). Individual Differences in Sensory and Consumer Science: Experimentation, Analysis and Interpretation.

[50] Racette C.M., Drake M.A. (2022). Consumer perception of natural hot-pepper cheeses. J. Dairy Sci..

[51] Hansen R., Gebhardt B., Hess S. (2023). Hype or hope? What consumer motives tell us about the prospects for plant and animal-based dairy products in six European countries. Food Qual. Prefer..

[52] Modlinska K., Adamczyk D., Maison D., Pisula W. (2020). Gender differences in attitudes to vegans/vegetarians and their food preferences, and their implications for promoting sustainable dietary patterns–a systematic review. Sustainability.

[53] Perez-Cueto F.J. (2020). Sustainability, health and consumer insights for plant-based food innovation. Int. J. Food Des..

[54] Marx J.A. (2012). “The Days Had Come of Curds and Cream” The origins and development of cream cheese in America, 1870–1880. Food Cult. Soc..

[55] Jeon S.-S., Lee S.-J., Ganesan P., Kwak H.-S. (2012). Comparative study of flavor, texture, and sensory in cream cheese and cholesterol-removed cream cheese. Food Sci. Biotechnol..

[56] Guinee T.P., Hickey M. (2009). Cream cheese and related products. Dairy Fats and Related Products.

[57] Wendin K., Langton M., Caous L., Hall G. (2000). Dynamic analyses of sensory and microstructural properties of cream cheese. Food Chem..

[58] Philadelphia Our Products. https://www.creamcheese.com/products.

[59] Soyinfo Center History of Cheese, Cream Cheese and Sour Cream Alternatives (with or without Soy) (1896–2013). https://www.soyinfocenter.com/books/169.

[60] Good Food Institute 2022 State of the Industry Report—Plant-Based Meat, Seafood, Eggs and Dairy. https://gfi.org/wp-content/uploads/2023/01/2022-Plant-Based-State-of-the-Industry-Report.pdf.

[61] GlobeNewswire Dairy-Free Cream Cheese Market Continues to Flourish with a Market Value of USD 300.40 Million by 2032 as Health-Conscious Consumers Embrace Plant-Based Alternatives. https://www.globenewswire.com/news-release/2023/08/22/2729705/0/en/Dairy-Free-Cream-Cheese-Market-Continues-to-Flourish-with-a-market-value-of-USD-300-40-million-by-2032-as-Health-Conscious-Consumers-Embrace-Plant-Based-Alternatives.html.

[62] Stats NZ Cheese Export Values Stretch to New Highs. https://www.stats.govt.nz/news/cheese-export-values-stretch-to-new-highs.

[63] Craig W.J., Mangels A.R., Brothers C.J. (2022). Nutritional profiles of non-dairy plant-based cheese alternatives. Nutrients.

[64] Fan M., Wei T., Lu X., Liu M., Huang Y., Chen F., Luo T., Fan Y., Liu R., Deng Z. (2023). Comprehensive quality evaluation of plant-based cheese analogues. J. Sci. Food Agric..

[65] Janhoj T., Frost M.B., Prinz J., Ipsen R. (2009). Sensory and Instrumental Characterization of Low-Fat and Non-Fat Cream Cheese. Int. J. Food Prop..

[66] Jaeger S.R., Roigard C.M., Jin D., Xia Y., Zhong F., Hedderley D.I. (2020). A single-response emotion word questionnaire for measuring product-related emotional associations inspired by a circumplex model of core affect: Method characterisation with an applied focus. Food Qual. Prefer..

[67] Kraft Foods Kitchens (1988). Philadelphia Cream Cheese Cookbook.

[68] Lumivero (2023). XLSTAT Statistical and Data Analysis Solution.

[69] Vidal L., Ares G., Jaeger S.R. (2021). Differences in citation proportions in CATA questions can be interpreted as differences perceived intensity of sensory attributes. J. Sensory Stud..

[70] Greenacre M. (2016). Correspondence Analysis in Practice.

[71] Meyners M., Castura J.C., Carr B.T. (2013). Existing and new approaches for the analysis of CATA data. Food Qual. Prefer..

[72] Beh E.J., Lombardo R. (2014). Correspondence Analysis: Theory, Practice and New Strategies.

[73] Hailpern S.M., Visintainer P.F. (2003). Odds ratios and logistic regression: Further examples of their use and interpretation. Stata J..

[74] Moskowitz H. (2020). Food and drink: Thoughts on base size in sensory and consumer research. Edelweiss Appl. Sci. Technol..

[75] Giacalone D., Clausen M.P., Jaeger S.R. (2022). Understanding barriers to consumption of plant-based foods and beverages: Insights from sensory and consumer science. Curr. Opin. Food Sci..

[76] Gorman M., Moss R., Fisher C., Knowles S., Ritchie C., Schindell K., McSweeney M.B. (2023). Perceptions of plant-based fish among Atlantic Canadians. J. Food Sci..

[77] Tso R., Lim A.J., Forde C.G. (2021). A critical appraisal of the evidence supporting consumer motivations for alternative proteins. Foods.

[78] Sogari G., Caputo V., Joshua Petterson A., Mora C., Boukid F. (2023). A sensory study on consumer valuation for plant-based meat alternatives: What is liked and disliked the most?. Food Res. Int..

[79] Chung Y.-L., Kuo W.-Y., Liou B.-K., Chen P.-C., Tseng Y.-C., Huang R.-Y., Tsai M.-C. (2022). Identifying sensory drivers of liking for plant-based milk coffees: Implications for product development and application. J. Food Sci..

[80] Muñoz A.M., Civille G.V. (1987). Factors affecting perception and acceptance of food texture by American consumers. Food Rev. Int..

[81] Moss R., LeBlanc J., Gorman M., Ritchie C., Duizer L., McSweeney M.B. (2023). A prospective review of the sensory properties of plant-based dairy and meat alternatives with a focus on texture. Foods.

[82] DataM Intelligence Vegan Frozen Pizza Market Size, Share, Industry, Forecast and Outlook (2023–2030). https://www.datamintelligence.com/research-report/vegan-frozen-pizza-market.

[83] Pointke M., Pawelzik E. (2022). Plant-based alternative products: Are they healthy alternatives? Micro- and macronutrients and nutritional scoring. Nutrients.

[84] Beauchamp G.K. (2016). Why do we like sweet taste: A bitter tale?. Physiol. Behav..

[85] Hetherington M.M., Bell A., Rolls B.J. (2000). Effects of repeat consumption on pleasantness, preference and intake. Br. Food J..

[86] Simons T., McNeil C., Pham V.D., Wang S., Wang Y., Slupsky C., Guinard J.-X. (2019). Chemical and sensory analysis of commercial Navel oranges in California. NPJ Sci. Food.

[87] Stolzenbach S., Bredie W.L.P., Christensen R.H.B., Byrne D.V. (2016). Understanding liking in relation to sensory characteristics, consumer concept associations, arousal potential and “appropriateness for use” using apple juice as an application. J. Sens. Stud..

[88] Martin N. (2002). Sweet/sour balance in champagne wine and dependence on taste/odour interactions. Food Qual. Prefer..

[89] Bhat S., Marklund M., Henry M.E., Appel L.J., Croft K.D., Neal B., Wu J.H.Y. (2020). A systematic review of the sources of dietary salt around the world. Adv. Nutr..

[90] Jervis M.g., Drake M.a. (2014). The use of qualitative research methods in quantitative science: A review. J. Sens. Stud..

[91] Oh M., Badu Baiden F., Kim S., Lema J. (2023). Identification of delighters and frustrators in vegan-friendly restaurant experiences via semantic network analysis: Evidence from online reviews. Int. J. Hosp. Tour. Admin..

[92] Silanikove N., Leitner G., Merin U. (2015). The Interrelationships between Lactose Intolerance and the Modern Dairy Industry: Global Perspectives in Evolutional and Historical Backgrounds. Nutrients.

[93] Michel F., Hartmann C., Siegrist M. (2021). Consumers’ associations, perceptions and acceptance of meat and plant-based meat alternatives. Food Qual. Prefer..

