# Supplementary material for: Plant-Based Alternatives Need Not Be Inferior: Findings from a Sensory and Consumer Research Case Study with Cream Cheese
Source: Foods. 2024 Feb 13;13(4):567. doi: 10.3390/foods13040567 (PMC10887787; doi:10.3390/foods13040567)
Supplement: Supplementary file 1 [file foods-13-00567-s001.zip › foods-2851570-supplementary.pdf]

SUPPLEMENTARY MATERIAL

PART 1. Supplementary sample information.

**Sample**	**PBCA1**	**PBCA2**	**Dairy1**	**Dairy2**
**Nutritional Information (average quantity per 100g)**				
Energy	1330kJ	1370kJ	940kJ	1490kJ
Calories	318	327	225	357
Protein, Total	Less than 1g	0.1g	4.9g	4.4g
Fat, Total	30.1g	30.8g	21.8g	35.4g
- Saturated	27.5g	28.2g	15.2g	23.4g
Carbohydrate	12.4g	13.2g	2.9g	6.1g
- Sugars	2.5g	2.6g	2.9g	5.2g
Sodium	855mg	879mg	350mg	411mg
**Ingredients**	Water, Coconut Oil, Modified Starch (1420, 1450, 1414), Sugar, Emulsifier (341, 450), Salt, Acid (270, 330), Acidity Regulator (331), Flavour, Thickener (415), Preservative (202), Colour (160a)	Water, Coconut Oil, Modified Pea & Maize Starch (1420, 1450), Sugar, Salt, Vegetable Gums (Carrageenan, Xantham Gum), Emulsifier (Calcium Phosphate), Acid (Lactic Acid, Citric Acid), Emulsifier (450), Acidity Regulator (331), Natural Flavour, Preservative (Potassium Sorbate)	Milk, Cream (From Milk), Milk Solids, Salt, Vegetable Gums (Locust Bean and/or Guar), Starter Culture, Processing Aids (Contain Milk)	Cream, Milk, Solids, Water, Mineral Salts (450, 452, 451, 339), Acid (Lactic Acid), Stabiliser (401), Salt
**Additional Information**	Non-GMO, no artificial colours or flavours		No artificial colours or flavours	
**Average Price (NZD/100g) as of October 2023**	$3.75	$3.29	$2.40	$2.08

PART 2. Image of samples as served to participants, in small glass bowl with piece of bread stick. A 3-digit random code was placed on the glass bowl to identify the sample.

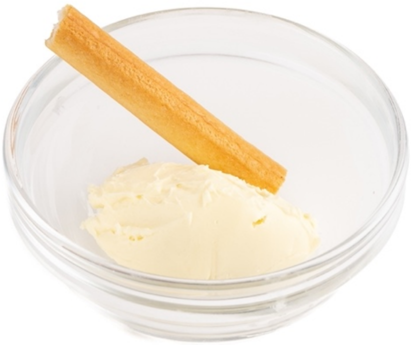

PART 3. Product characterisations for cream cheese by sensory and non-sensory descriptors based on responses from 157 participants (total sample). Of the four samples, two were plant-based (PBCA1 and PBCA2) and two were dairy (Dairy1 and Dairy2). Values are citation proportions (between 0 and 1). Significant differences between samples were established following Cochran's Q test (5% level) unless indicated by '(ns)' in descriptor name. **A)** Sensory descriptors, **B)** Emotional descriptors, **C)** Conceptual descriptors, **D)** Situational use descriptors.

A) Sensory

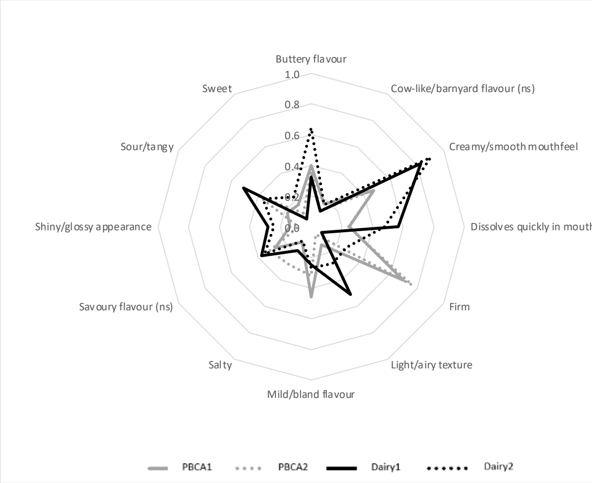

B) Emotional

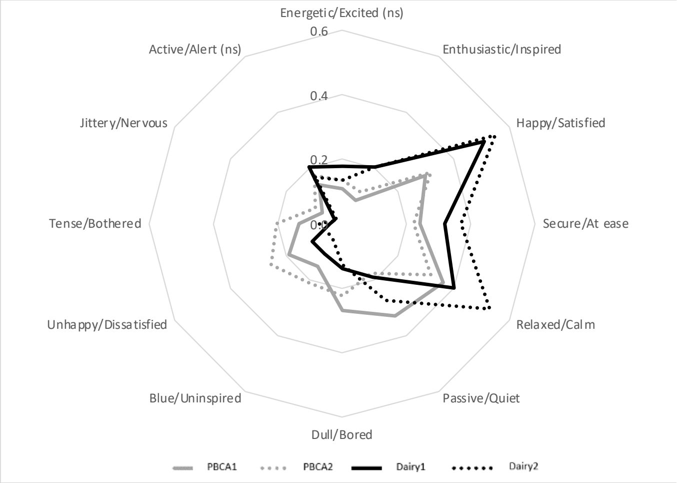

C) Conceptual

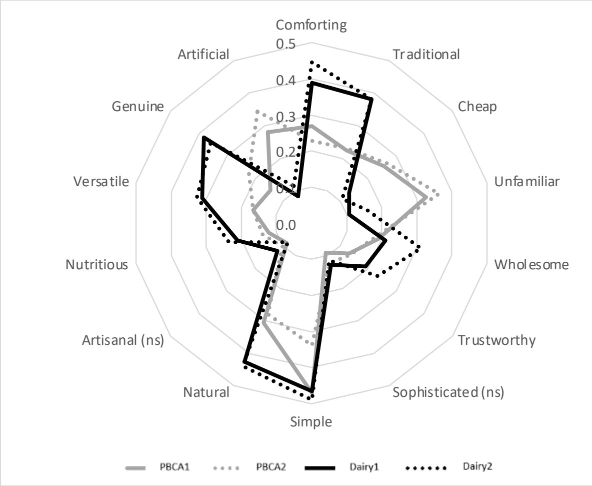

D) Situational use

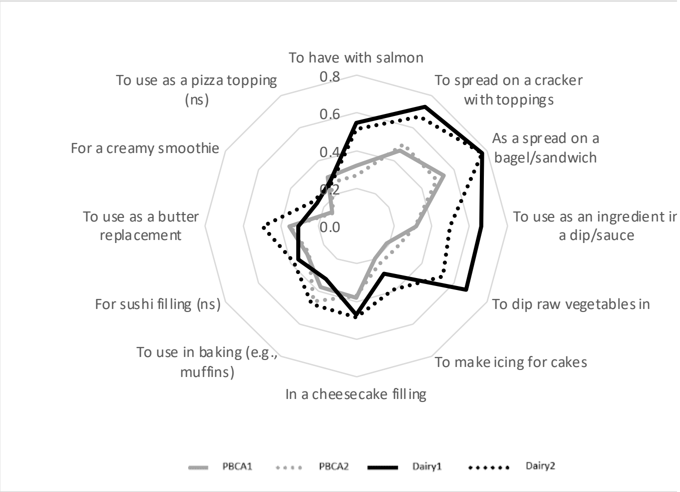

PART 4. Plots of the first two dimensions after Correspondence Analysis based on data from total sample (n=157), showing average sample positions with 95% confidence ellipses. Four samples of cream cheese were included in the study, where two were plant-based (PBCA) and two were dairy (from cow's milk). **A)** Sensory descriptors, **B)** Emotional descriptors, **C)** Conceptual descriptors, **D)** Situational use descriptors.

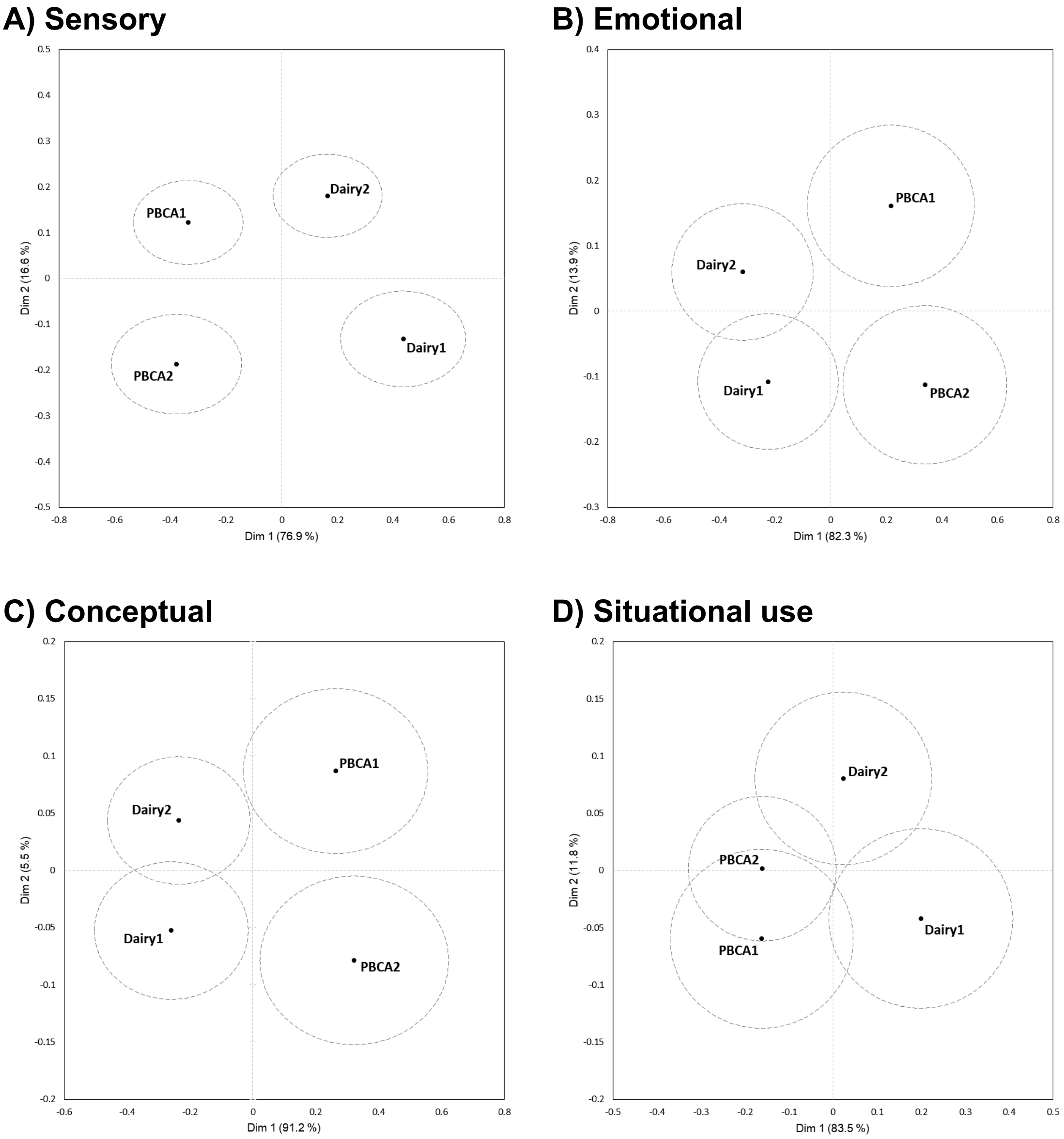

PART 5. Plot of the first two dimensions after Correspondence Analysis based on data from *PBCA Likers* cluster (n=111), showing average sample positions with 95% confidence ellipses based on sensory descriptors. Four samples of cream cheese were included in the study, where two were plant-based (PBCA) and two were dairy (from cow's milk).

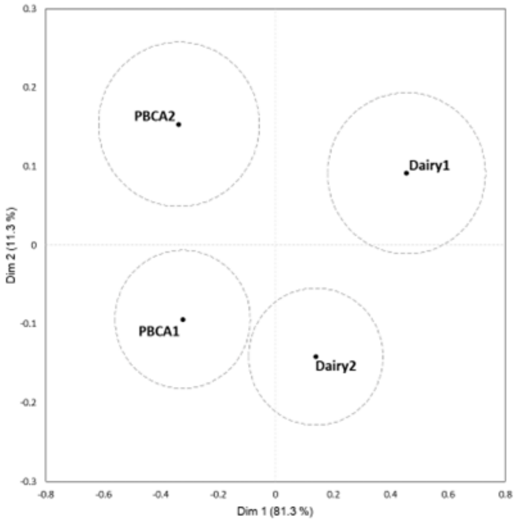

PART 6. Drivers of liking for cream cheese. Results for sensory and non-sensory terms (rows) in the *PBCA Dislikers* cluster (n=41). Column 2 gives the p-value from Cochran's Q test for sample differences, Column 3 gives the average term citation frequency (%) across all samples in the study, and Column 4 gives the mean impact on liking[$] when term was selected. Results must be regarded with caution due to low sample size and regarded as indicative only. For the same reason, significance testing on mean impact was not performed.

Response type and term	P-value from Cochran's Q test	Average citation frequency (%)	Mean impact on liking
**Sensory**			
Shiny/glossy appearance	0.005	17.1	1.0
Cow-like/barnyard flavour	0.456	12.2	0.3
Buttery flavour	0.0002	35.4	1.5
Mild/bland flavour	0.001	35.4	-0.8
Salty	0.002	20.1	-0.4
Savoury flavour	0.007	32.9	0.8
Firm	<0.0001	48.2	-1.9
Sweet	<0.0001	13.4	1.5
Sour/tangy	0.001	31.1	-0.5
Creamy/smooth mouthfeel	<0.0001	53.7	2.4
Dissolves quickly in mouth	<0.0001	27.4	1.5
Light/airy texture	<0.0001	20.7	1.9
**Emotional**			
Energetic/Excited	0.056	6.7	2.1
Enthusiastic/Inspired	0.002	8.5	1.8
Happy/Satisfied	<0.0001	35.4	2.8
Secure/At ease	<0.0001	22.6	2.1
Relaxed/Calm	<0.0001	32.3	2.0
Passive/Quiet	0.003	19.5	0.3
Dull/Bored	0.000	23.2	-1.7
Blue/Uninspired	<0.0001	19.5	-2.1
Unhappy/Dissatisfied	<0.0001	24.4	-3.1
Tense/Bothered	<0.0001	16.5	-2.5
Jittery/Nervous	0.001	7.9	-2.2
Active/Alert	0.026	11.0	1.8
**Conceptual**			
Comforting	<0.0001	27.4	2.3
Traditional	0.000	23.8	1.8
Cheap	<0.0001	29.9	-2.4
Unfamiliar	<0.0001	34.8	-2.5
Wholesome	<0.0001	16.5	2.1
Trustworthy	0.001	12.8	2.3
Sophisticated	0.245	6.7	1.3
Simple	0.010	40.9	1.1
Natural	0.000	31.1	1.6
Artisanal	0.957	8.5	0.8
Nutritious	0.007	12.2	1.7
Versatile	0.002	20.7	1.3
Genuine	<0.0001	20.7	2.3
Artificial	<0.0001	26.2	-2.5
**Situational**			

To have with salmon	<0.0001	32.9	1.9
To spread on a cracker with toppings	<0.0001	48.8	2.1
As a spread on a bagel/sandwich	<0.0001	50.0	2.5
To use as an ingredient in a dip/sauce	<0.0001	42.1	1.6
To dip raw vegetables in (e.g., carrot, cucumber)	<0.0001	37.2	2.1
To make icing for cakes	0.687	22.6	0.4
In a cheesecake filling	0.057	34.1	1.1
To use in baking (e.g., muffins)	0.132	38.4	0.0
For sushi filling	0.017	27.4	1.5
To use as a butter replacement	0.709	29.9	0.7
For a creamy smoothie	0.253	16.5	0.5
To use as a pizza topping	0.164	21.3	1.6

Notes.
[$]) 9-point liking response scale: 1 = dislike extremely, 9 = like extremely.
[#]) Collected as part of CATA question relating to sustainability, but placed in emotional and conceptual sections to capture the type of concepts these statements represent.
